# Interleukin 34 Serves as a Novel Molecular Adjuvant against *Nocardia Seriolae* Infection in Largemouth Bass (*Micropterus Salmoides*)

**DOI:** 10.3390/vaccines8020151

**Published:** 2020-03-28

**Authors:** Huy Hoa Hoang, Pei-Chi Wang, Shih-Chu Chen

**Affiliations:** 1 Department of Veterinary Medicine, College of Veterinary Medicine, National Pingtung University of Science and Technology, No. 1 Shuefu Road, Neipu, Pingtung 91201, Taiwan; hoanghuyhoa2003@yahoo.com (H.H.H.); pc921003@gmail.com (P.-C.W.); 2 Southern Taiwan Fish Disease Centre, National Pingtung University of Science and Technology, No. 1 Shuefu Road, Neipu, Pingtung 91201, Taiwan; 3 International Degree Program of Ornamental Fish Science and Technology, International College, National Pingtung University of Science and Technology, No. 1, Shuefu Road, Neipu, Pingtung 91201, Taiwan; 4 Research Centre for Animal Biologics, National Pingtung University of Science and Technology, Pingtung 91201, Taiwan

**Keywords:** Nocardiosis, *Nocardia seriolae*, Interleukin-34, DNA vaccine, molecular adjuvant

## Abstract

DNA vaccines have been widely employed in controlling viral and bacterial infections in mammals and teleost fish. Co-injection of molecular adjuvants, including chemokines, cytokines, and immune co-stimulatory molecules, is one of the potential strategies used to improve DNA vaccine efficacy. In mammals and teleost fish, interleukin-34 (IL-34) had been described as a multifunctional cytokine and its immunological role had been confirmed; however, the adjuvant capacity of IL-34 remains to be elucidated. In this study, IL-34 was identified in largemouth bass. A recombinant plasmid of IL-34 (pcIL-34) was constructed and co-administered with a DNA vaccine encoding hypoxic response protein 1 (Hrp1; pcHrp1) to evaluate the adjuvant capacity of pcIL-34 against *Nocardia seriolae* infection. Our results indicated that pcIL-34 co-injected with pcHrp1 not only triggered innate immunity and a specific antibody response, but also enhanced the mRNA expression level of immune-related genes encoding for cytokines, chemokines, and humoral and cell-mediated immunity. Moreover, pcIL-34 enhanced the protection of pcHrp1 against *N. seriolae* challenge and conferred the relative percent survival of 82.14%. Collectively, IL-34 is a promising adjuvant in a DNA vaccine against nocardiosis in fish.

## 1. Introduction

Nocardiosis, caused by the facultative intracellular bacteria *Nocardia seriolae*, is a problematic infection in the fish aquaculture industry and leads to severe economic loss in Asian countries, especially Taiwan, China, Japan, and Vietnam [1,2,3,4,5]. Reportedly, nocardiosis infects various marine and freshwater fish species in Taiwan, including largemouth bass (*Micropterus salmoides)*, grouper (*Epinephelus* sp.), snubnose pompano (*Trachinotus blochii*), crimson snapper (*Lutjanus erythropterus*), and spotted scat (*Scatophagus argus*) [6]. Nocardiosis treatment relies mainly on chemical and pharmaceutical therapies; however, the use of these compounds can lead to contamination of the aquatic environment, accumulation of antibiotics in the fish body, and appearance of drug-resistance bacteria. Therefore, a safe and efficacious vaccine that could provide long-term protection and environmental friendliness is urgently needed for the sustainable development of the aquaculture industry.

A DNA vaccine encompasses a gene delivered from pathogenic viruses or bacteria, encoding an immunogenic protein and a vector with a eukaryotic cell promoter, to help drive protein expression. Compared with traditional inactivated and subunit vaccines, DNA vaccines have been documented to stimulate a robust cytotoxic T cell response. Furthermore, DNA vaccines can induce both humoral and cellular adaptive immune responses by evolving with major histocompatibility complex (MHC) class I and MHCII antigen presentation by dendritic cells [7,8]. Recent studies have shown that DNA vaccines could be used to control viral pathogens; however, only a few have been tested against bacterial infections. Reportedly, DNA vaccines have been utilized in fish to control *Streptococcus iniae* [9], *Vibrio anguillarum* [10,11], *Edwardsiella tarda* [12,13,14], and *Nocardia seriolae* [15,16], indicating that DNA vaccines could be suitable for controlling bacterial diseases by provoking both non-specific and specific immune host responses. Despite the advantages of DNA vaccines, including low cost, safety, stability, and efficacy against bacterial and viral diseases, there are only two DNA vaccines licensed for use in commercial aquaculture. In 2005, APEX-IHN (Novartis/Elanco) was the first DNA vaccine licensed for protecting Atlantic salmon against the infectious hematopoietic necrotic virus (IHNV) [17]. CLYNAV (Elanco), a polyglycoprotein-encoding DNA vaccine against salmon pancreas disease virus (SPDV) infection, was the second DNA vaccine approved in 2016 for aquaculture [18]. However, the availability of these two licensed DNA vaccines is not reassuring for the numerous microorganism diseases occurring in the aquaculture industry. Currently, researchers are focused on improving the immunogenicity of DNA vaccines, including codon optimization, co-vaccination, or construction of bicistronic DNA vaccines with molecular adjuvants [19]. Among these strategies, the co-injection of plasmid DNA that encodes cytokines or chemokines is a promising option for enhancing the efficacy of DNA vaccines due to their role in modulating the host immune response to infection.

Cytokines consist of a large group of proteins, peptides, or glycoproteins that are involved in a broad range of immune responses, from the induction of both innate and adaptive immunity to the generation of cytotoxic T cells, and defense against microorganism infections [20]. Cytokines are classified into different families, including interleukins (IL), chemokines, colony-stimulating factors (CSF), interferons (IFN), transforming growth factors (TGF), and tumor necrosis factors (TNF). In recent years, the identification of several cytokine genes has been documented in teleost species. Nevertheless, the number of studies that focus on the application of cytokine genes as molecular adjuvants in fish is limited when compared to those undertaken in mammals [21]. To date, it has been reported that fish cytokines such as IL-6 [22], IL-8 [23,24,25], IL-2 [24,26], IL-1β [24,25,27], IL-15, IL-17 [24], TNFα, granulocyte colony-stimulating factors (G-CSF) [25], IL12 [28], chemokine (C-C motif) ligand 3 (CCL3), CCL4, CCL19, CCL21 [29], and IFNγ [30] can be applied as promising molecular adjuvants for DNA or recombinant subunit vaccines against particular viral or bacterial infections. Interleukin-34 (IL-34), a cytokine newly discovered in 2008, has been documented as a pluripotent cytokine that modulates a wide array of cellular processes, including differentiation, adhesion, proliferation, survival, metabolism, inflammation, and immune modulators in mammals [31]. Currently, some IL-34 genes have been identified in teleost fish, including fugu, rainbow trout, zebrafish, Atlantic salmon, catfish, grouper, large yellow croaker, and grass carp [32,33,34,35]. Recently, grass carp IL-34 has demonstrated a pivotal role in modulating macrophage function and inflammation processes [35]. However, the adjuvanticity of IL-34 has not been achieved in these teleost species.

In the present work, the sequence and phylogenetic analysis of largemouth bass IL-34 (LMB-IL-34) were performed. Furthermore, we investigated the adjuvant effects of LMB-IL-34 by co-administration with a DNA vaccine encoding hypoxic response protein 1 (Hrp1) against *Nocardia seriolae* infection. Moreover, several aspects of immunity were evaluated, including specific antibody production, serum lysozyme activity, the expression of immune-related genes, and protective immunity.

## 2. Materials and Methods 

### 2.1. Ethics Statement

All animal experiments were reviewed and approved by the Institutional Care and Use Committee (IACUC), National Pingtung University of Science and Technology (Protocol # NPUST-106-068).

### 2.2. Fish, Bacterial Strains, Plasmids, Reagents, and Growth Conditions

Healthy largemouth bass (55 ± 5 g) purchased from an aquaculture farm in Pingtung, Taiwan, were maintained in aquarium tanks consisting of three 3500 L tanks with a circulatory system. The water temperature was consistently maintained at 28 °C, and the fish were acclimatized to laboratory conditions for two weeks before conducting experiments. The fish were fed daily with commercial pellets. Before performing the experiments, five fish were sacrificed and subjected to bacterial isolation and polymerase chain reaction (PCR) analysis of the head kidney, spleen, and liver to confirm that fish were not infected with *N. seriolae* [36].

The virulent *N. seriolae* strain 961113 was originally isolated from diseased striped bass and maintained in our laboratory. The strain was cultured on brain–heart infusion (BHI, Becton Dickinson, Le Pont-de-Claix, France) for 3 days at 25 °C. *Escherichia coli* DH5α (Genemark, Taichung, Taiwan) was used as the host strain for cloning, cultivated in Luria–Bertani medium (LB, Becton Dickinson, Le Pont-de-Claix, France) supplemented with 100 µg/mL ampicillin (Bio Basic, Ontario, Canada) at 37 °C. The plasmid pcDNA3.1(+) (Invitrogen, Carlsbad, CA, USA) was used for the eukaryotic expression of IL-34 and Hrp1 genes.

### 2.3. Sequence and Phylogenetic Analysis

The sequence analysis of the open reading frame (ORF) of the IL-34 gene was conducted using the ORF Finder [37]; nucleotide/protein translation and molecular weight predictions were predicted using the Translation Tools and Compute pI/Mw tools on the ExPASy server [38]. The signal peptide was predicted with the SignalP-5.0 online tool [39]. The N-glycosylation sites were predicted using the N-GlydDE web server [40]. The Basic Local Alignment Search Tool (BLAST) program [41] was used for homology search, and the identity of the IL-34 amino acid sequences was calculated using the MegAlign program (DNASTAR 5.0, DNASTAR Inc, Madison, Wisconsin). The phylogenetic tree was constructed using the neighbor-joining method (1000 bootstraps) with the Molecular Evolutionary Genetics Analysis (MEGA) 7.0 program.

### 2.4. Plasmid Construction and Cloning of pcIL-34 and pcHrp1

To maximize the transcription and translation capability of Hrp1 and IL-34 genes in a eukaryotic expression system, the Kozak consensus sequences (GCCACCATGG) [42] were inserted in the forward primers of each gene. In addition, a 6 × histidine tag sequence was also added in the reverse primers to facilitate the evaluation of IL-34 and Hrp1 expression in vitro and in vivo. 

Genomic DNA from the *N. seriolae* strain 961113 was extracted using the Genomaker® DNA extraction kit (Bio-East Technology, Taipei, Taiwan) following the manufacturer’s instructions. The primers Hrp1F/Hrp1R containing *Nhe* I and *Hind* III restriction enzyme cutting sites (Table 1), respectively, were designed using the Hrp1 gene sequence from our previous study [43]. PCR amplification was performed under the following thermal parameters: 95 °C for 5 min, 35 cycles of 95 °C for 1 min, 65 °C for 45 sec, 72 °C for 1 min, followed by 72 °C for 10 min. The PCR products were identified on a 1% agarose gel and purified using a gel purification system (Favorgen, Pingtung, Taiwan). The purified PCR product was digested with the *Nhe I* and *Hind III* restriction enzymes (New England Biolab, Hitchin, UK) and inserted into pcDNA3.1 (+) to generate the recombinant plasmid pcDNA3.1/Hrp1 (pcHrp1). The recombinant plasmid pcHrp1, containing the Hrp1 open reading frame, was transformed into *E. coli* DH5α cells and then sequenced by Genomics BioSci and Tech Co. Ltd (New Taipei, Taiwan).

Total RNA was isolated from the head kidney of healthy largemouth bass using Zymeset reagent (Everything Biotech, New Taipei, Taiwan). Extracted RNA samples were treated with DNase I (New England Biolabs, Hitchin, UK) to eliminate genomic DNA contamination as described previously [44]. The quality and quantity of isolated RNA samples were then confirmed using a Nanophotometer (Implen, München, Germany), and then, cDNA was synthesized from 1 µg of total RNA using an iScript™ cDNA Synthesis Kit (Bio-Rad, Irvine, CA, USA) according to the manufacturer’s instructions, and was stored at −20 °C until used. The largemouth bass IL-34 gene sequence was retrieved from our transcriptomic data [45] and amplified by PCR using the cDNA template prepared above and primers IL34F/IL34R (Table 1). The specific primers of IL-34 were designed by adding the restriction enzyme sites *Hind III* and *EcoR I* at the 5’ of the forward and reverse primer. The PCR conditions were identical to those described above. The PCR product was analyzed on a 1% agarose gel, purified, digested with the corresponding restriction enzymes, ligated, cloned into pcDNA3.1(+) to generate recombinant plasmid IL-34 (pcIL-34), transformed into *E. coli* DH5α, and sequenced as previously described.

### 2.5. In vitro Expression Analysis of pcIL-34 and pcHrp1

The in vitro expression of the Hrp1 and IL-34 genes from the recombinant plasmid DNA was confirmed by the transfection of the E11 cell line (derived from snakehead fish). The E11 cell line was kindly provided by Duc Tan Nguyen (Institute of Veterinary Research and Development of Central Vietnam, Nhatrang, Vietnam). The E11 cell line has been successfully applied for both stable transfection and rescue of recombinant virus [46]. Briefly, the E11 cells were cultured in L-15 medium (Invitrogen, Carlsbad, CA, USA), containing 10% fetal bovine serum (FBS) (Invitrogen, USA), 100 IU/mL penicillin and 100 μg/mL streptomycin (Corning Inc, Corning, NY, USA). Four micrograms of each DNA plasmid were transfected per well into E11 cells in 6-well culture plates with Lipofectamine 2000 (Invitrogen, Carlsbad, CA, USA) following the manufacturer’s instructions; empty plasmid pcDNA3.1(+) was used as the negative control. The cells were harvested 72 h after transfection and subjected to Western blot analysis.

### 2.6. Vaccination and Sampling 

The DNA plasmids (pcIL-34, pcHrp1, and pcDNA3.1) were extracted from *E. coli* DH5α using the Endotoxin-Free Ultrapure Plasmid Extraction Miniprep System (Viogene, New Taipei, Taiwan) according to the manufacturer’s instructions. The concentration and purity of the plasmid DNA were confirmed using a nanophotometer (Implen, München, Germany), and then diluted in phosphate-buffered saline (PBS) to a concentration of 250 µg/mL according to recent studies [16,23,47]. pcIL-34+pcHrp1 was obtained by mixing pcIL-34 with an equal volume of pcHrp1.

In total, 565 largemouth bass were randomly divided into five groups (113 fish per group) and reared separately in the same aquarium as described above. The fish were injected intramuscularly (i.m.) with 0.1 mL of PBS, pcDNA3.1, pcIL-34, pcHrp1, and pcHrp1+pcIL-34. At 7 days, 14 days, 21 days, and 28 days post-vaccination (p.v.), blood samples were randomly collected from five fish in each experimental group using the caudal vein and were allowed to clot at 4 °C overnight for serum isolation. After blood collection, fish were euthanized using an overdose of 2-phenoxyethanol (C_6_H_5_0CH_2_CH_2_0H; First Chemical Works, Taipei, Taiwan). The serum was collected for the evaluation of serum lysozyme activity and specific-antibody responses. The head kidney of eight fish in each group were individually collected for qRT-PCR analysis of immune-related genes 24 h post-challenge (p.c) on 28-day p.v.

### 2.7. Challenge

At 28 days post-vaccination, 60 fish from each experimental group were randomly divided into two sub-groups with 30 fish each and maintained in separate compartments of the same aquarium as described above. Fish were anesthetized and challenged with 0.1 mL of 5.5 × 10^6^ colony-forming units (cfu)/mL of *N. seriolae*. Mortality was recorded daily for 40 days after challenge, and bacteria were isolated from the mesentery, head kidney, liver, and spleen of dead and moribund fish to confirm the presence of inoculated *N. seriolae*. Furthermore, the relative percent of mortality (RPS) was calculated using the following formula:

$$\mathrm{RPS}=\left(1-\left(\frac{\%\;\mathrm{mortality}\;\mathrm{of}\;\mathrm{vaccine}\;\mathrm{group}}{\%\;\mathrm{mortality}\;\mathrm{of}\;\mathrm{control}\;\mathrm{group}}\right)\right)\times 100$$

### 2.8. Detection of pcIL-34 and pcHrp1 in the Muscle of Immnunized Fish by PCR

In each experimental group, muscle tissue at the injection site of five fish was collected at 7 days, 14 days, 21 days, and 28 days p.v.; next, DNA was isolated from muscle tissues using the Genomaker® DNA extraction kit (Bio-East Technology, Taipei, Taiwan) following the manufacturer’s instructions and stored at −20 °C until PCR detection. PCR was performed using the specific primers Hrp1-F/Hrp1-R2 and IL-34-F/IL-34-R2 (Table 1) for Hrp1 and IL-34 gene detection, respectively. 

### 2.9. In Vivo Transcription Analysis of pcIL-34 and pcHrp1 by RT-PCR

At 21 days p.v., five fish from each experimental group were sacrificed to collect muscle tissue. Total RNA from muscle tissue was isolated using Zymeset reagent (Everything Biotech, New Taipei, Taiwan), treated with DNase I, and synthesized into cDNA as described above. Hrp1 and IL-34 gene segments were amplified from the synthesized cDNA with primer set as described above (Table 1). The Rsp40 gene was used as an internal control.

### 2.10. In vivo Expression Analysis of pcIL-34 and pcHrp1 by Western Blotting

Expression of pcIL-34 and pcHrp1 in largemouth bass muscle tissue was confirmed by Western blotting at 21 days p.v. as described previously [48,49]. The muscle tissue of five fish in each experimental group were biopsied at the injection site, homogenized in buffer containing 100 mmol mL^−1^ Tris-HCl, pH = 6.8; 1.0 mmol mL^−1^ phenylmethysulfonyl fluoride (PMSF), 6% Sodium dodecyl sulfate (SDS) and 2% β-mercapthoethanol, and then subjected to a 12% SDS-PAGE gel. Muscle tissues collected from PBS and pcDNA3.1 groups were used as negative controls.

### 2.11. Western Blot Analysis

The Western blot analysis was performed to confirm the in vitro and in vivo expression of plasmid DNA as previously described [50]. Briefly, the transfected cell lysate and muscle tissue samples were mixed with 4 × sample buffer, then separated by 12% SDS-PAGE gel and transferred to polyvinylidene difluoride (PVDF) membranes (Invitrogen, USA) at 12 V for 1 h. The membrane was incubated with 5% skim milk (Fronterra, Aukland, New Zealand) in 0.1% Tween-20 in PBS (PBST) overnight at 4 °C to prevent non-specific binding. Next, the mouse anti-6-histidine antibody (Merck, Darmstadt, Germany) (diluted at 1:1000 in PBST) was applied to the membrane and incubated at 4 °C for 12 h. The rabbit anti-mouse IgG-horseradish peroxidase (1:3000 dilution in PBST; Santa Cruz Biotechnology, Dallas, TX, USA) was added to the membrane for 1 h at 25 °C. The membrane was washed five times with PBST before every successive step. After the final washes, the membranes were visualized using Western Lightning^TM^ Plus-ECL (PerkinElmer Inc., Waltham, MA, USA) using the Luminescence Fluorescence Imaging System (Syngene, Frederick, MD, USA).

### 2.12. Serum Lysozyme Activity

The serum lysozyme activity was measured using the turbidimetric method as described by Ellis [51] and Hoang et al [43]. Briefly, 180 µL of *Micrococcus lysodeikticus* at a concentration of 0.02% (*w*/*v*) in 0.05 M phosphate buffer solution at pH = 6.2, was mixed with 20 µL of fish serum at 25 °C. After adding *M. lysodeikticus*, the optical density (OD) was measured using a spectrophotometer (450 nm) at 0.5 min and 5.5 min, respectively. Each test was performed in triplicate.

### 2.13. Enzyme-Linked Immunosorbent Assay (ELISA)

The production of a specific anti-rHrp1 antibody was analyzed by ELISA. The detailed protocol was well documented previously by Hoang et al [43]. Briefly, rHrp1 was diluted to a concentration of 5 µg/mL in PBS. Each well of a Nunc-Immuno 96 MicroWell solid plate (Sigma, St. Louis, MO, USA) was coated with 100 µL of diluted rHRP1 as the coating antigen, and sera from immunized and control groups (1:100 dilution in PBST) were added into the plates at 100 µL/well. The test was conducted in triplicate.

### 2.14. qRT-PCR Analysis of the Expression of Immune-Related Genes

The head kidneys of eight fish were collected from experimental fish at 24 h post-challenge. Total RNA extraction and cDNA synthesis were performed as described above. qRT-PCR was performed to investigate the expression of immune-related genes, including pro-inflammatory cytokines (IL-1β, TNFα, and IL-6), cell mediated-immunity mediators (MHCIα, cluster of differentiation (CD)8α, IL-12p40, IL-18, and IFNγ), humoral immunity mediators (MHCIIα, CD4-1, and immunoglobulin (Ig)M), transcriptional activators (signal transducer and activator of transcription (STAT)1, STAT3, and nuclear factor-kappa light chain enhancer of activated B cells (NF-κB)), and chemokines (IL-8, chemokine (C-C motif) ligand (CCL)2, CCL20, chemokine (C-X-C motif) ligand (CXCL)9, CXCL10). All immune-related gene sequences were retrieved based on our data from previous studies [43,45] and primers for qRT-PCR were designed as listed in Table 1. Each assay was conducted in triplicate with the ribosomal protein Rsp40 and β-actin as the reference genes for normalization as previously described [43,45,52]. The qRT-PCR was performed using the iQ^TM^ SYBR Green Supermix (Bio-Rad, Hercules, CA, USA) in a CFX96 Real-Time PCR detection system (Bio-Rad Laboratories Inc., Hercules, CA, USA). RT-PCR was conducted with the following conditions: 95 °C for 3 min, followed by 40 cycles of 95 °C for 15 s, and 59 °C for 30 s. The threshold cycle (Ct) value was calculated automatically using the CFX Manager^TM^ Software, version 3.1 (Bio-Rad, Hercules, CA, USA). The relative expression levels of these immune-related genes were analyzed using the 2^−ΔΔCt^ method previously described by Livak et al. (2001) [53].

### 2.15. Statistical Analysis

Statistical analysis was performed using the SPSS 22 software (SPSS Inc, Chicago IL, USA) to compare differences among experimental groups by one-way analysis of variance (ANOVA) with post-hoc Duncan’s multiple range test (DMRT). The significance level was defined as *p* < 0.05 and all data are expressed as mean ± standard deviation (SD) values.

## 3. Results

### 3.1. Sequence Analysis of IL-34

**Figure 1:**
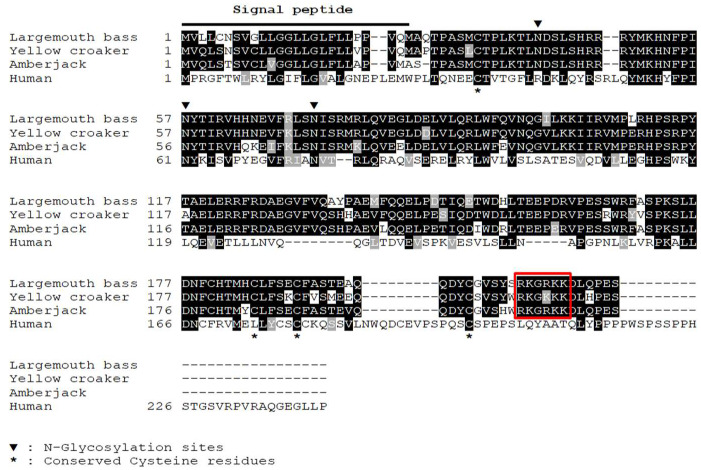
Amino acid sequence alignment of largemouth bass (LMB)-IL-34 in this study with reference IL-34s deposited in NCBI. The line above the alignment presented the signal peptides, the N-glycosylation sites are indicated by a triangle above the alignment, the conserved cysteine residues are indicated by a star below, and the lysine/argine rich motifs (RKG[R/K]K) are shown in the red box.

The ORF of LMB-IL-34 was 657 bp which encodes a 213 deduced amino acid (a.a.) sequence, with a theoretical molecular mass of 25.39 kDa. The first 25 a.a. were predicted as signal peptides and three potential N-glycosylation sites were found in the LMB-IL-34 sequence. The multiple sequence alignment revealed that four of six conserved mammalian cysteine residues were found in LMB-IL-34. These N-glycosylation sites and cysteine residues are reportedly crucial for the formation of intramolecular disulfide bonding, stability, and proper folding of human IL-34 [54]. The C-terminal tail of LMB-IL-34 is about 29 a.a. shorter than human IL-34, which contains an enriched region of proline-serine-threonine (Pro-Ser-Thr), a typical characteristic of flexible mucin-like O-linked glycosylation-rich sequences that are absent in teleost species [55]. However, the C-terminal tail of LMB-IL-34 and other fish IL-34s comprised a conserved cationic lysine/arginine-rich motif (RKG[R/K]K) not observed in human IL-34 (Figure 1).

**Figure 2:**
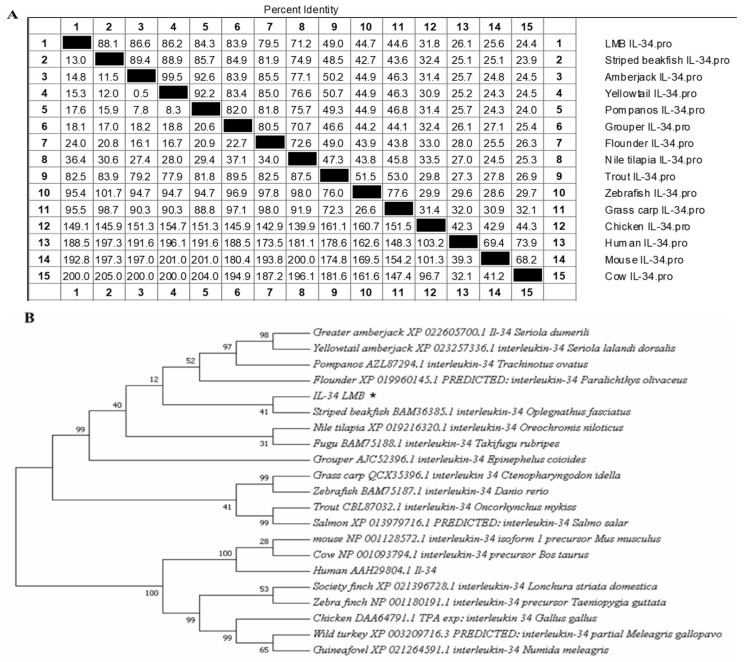
(**A**) Multiple sequences alignment of LMB-IL-34 with other reference IL-34s. (**B**) Phylogenetic tree of LMB-IL-34 with other reference IL-34s. The number at each branch represents the bootstrap values obtained with 1000 replicates. (*) indicates the LMB-IL-34 in this study.

The identity of the IL-34 a.a. sequence between LMB and other species is shown in Figure 2A. LMB-IL-34 shared 24.9–88.1% identities with other known homologs. LMB-IL-34 shared high similarities with striped beakfish IL-34, amberjack IL-34, yellowtail IL-34, and pompano IL-34, respectively (88.1%, 86.6%, 86.2%, and 84.3%). Fish IL-34s are highly conserved, sharing 45.1–88.1% a.a. identity. The phylogenetic analysis revealed that LMB-IL-34 was clustered into a separate group apart from mammalian and bird IL-34s. Furthermore, LMB-IL-34 formed a separate cluster with the IL-34 of grass carp, zebrafish, trout, and salmon (Figure 2B).

### 3.2. Cloning and In Vitro Expression of Plasmid pcIL-34 and pcHrp1

**Figure 3:**
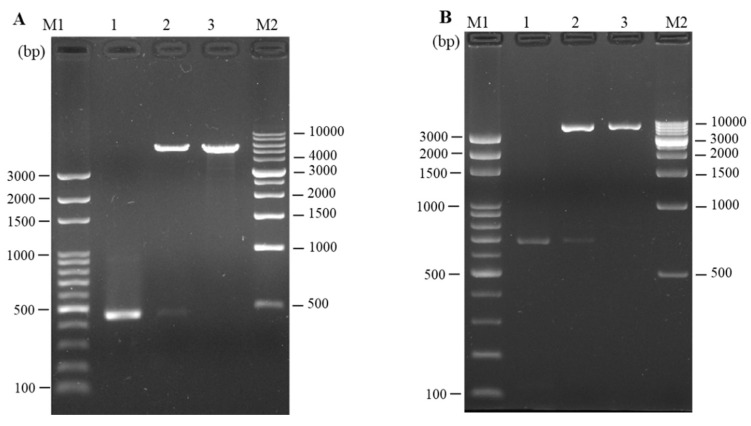
(**A**) Identification of recombinant plasmid pcHrp1 with PCR and enzyme digestion; M1: DNA marker (100 bp); lane 1: PCR product of Hrp1 gene (459 bp); lane 2: digestion of plasmid pcHrp1 with *Nhe I* and *Hind III*; lane 3: digestion of plasmid Hrp1 with *Nhe I*; M2: DNA marker (1000 bp). (**B**) Identification of recombinant plasmid pcIL-34 with PCR and enzyme digestion; M1: DNA marker (100 bp); lane 1: PCR product of IL-34 gene (693 bp); lane 2: digestion of plasmid pcIL-34 with *Hind III* and *EcoR I*; lane 3: digestion of plasmid Hrp1 with *HindIII*; M2: DNA marker (1000 bp).

**Figure 4:**
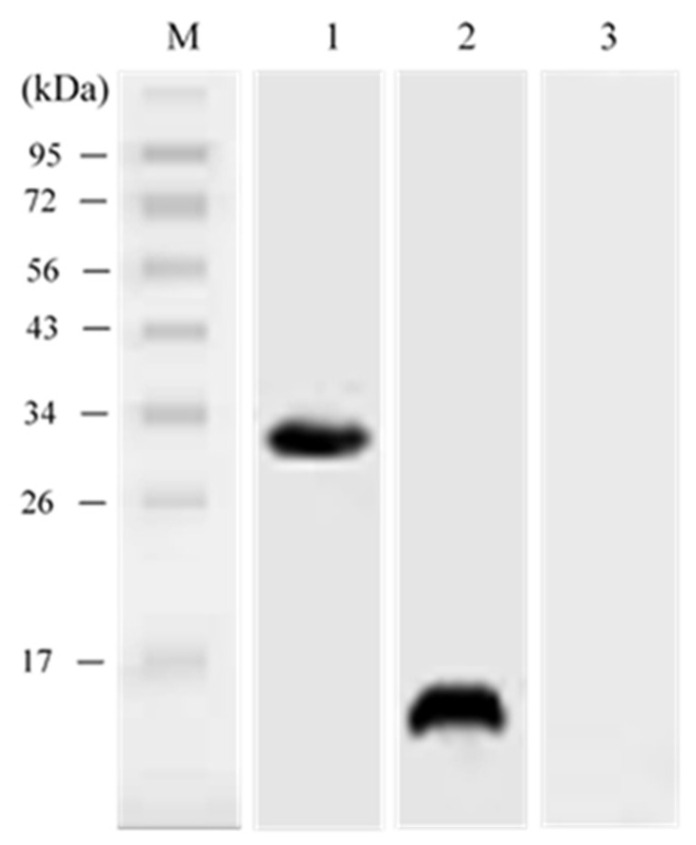
In vitro detection of pcHrp1 and pcIL-34 by Western blot. Lane M: protein ladder; lane 1: lysate of E11 cells transfected with pcIL-34; lane 2: lysate of E11 cells transfected with pcHrp1; lane 3: lysate of E11 cells transfected with pcDNA3.1 (negative control).

The full length of LMB-IL-34 and the *N. seriolae* Hrp1 gene was successfully amplified using PCR with 693 bp and 459 bp products, respectively, which included a complete ORF, Kozak sequences at the N-terminus, and 6 × histidine tag sequences at the C-terminus of each gene. The target DNA fragments were then cloned into pcDNA3.1(+) for eukaryotic expression. Enzyme digestion was performed to confirm that the recombinant plasmids contained IL-34 and Hrp1 DNA fragments (Figure 3A,B). Western blotting was conducted by using a monoclonal anti-histidine antibody to confirm the eukaryotic expression of pcIL-34 and pcHrp1. The recombinant plasmid DNA (pcIL-34, pcHrp1) was transfected into E11 cells; pcDNA3.1(+) was used as the negative control. Cells were collected at 72 h post-transfection and then subjected to Western blotting. The Western blot analysis of the cells transfected with pcIL-34 showed a clear and specific band, approximately 30 kDa, which was about 4.6 kDa higher than the predicted molecular weight of LMBIL-34 (25.39 kDa). Similarly, a protein of approximately 15 kDa was detected in the cells transfected with pcHrp1, corresponding to the theoretical molecular weight of the Hrp1 protein. No detectable band was observed in the cells transfected with pcDNA3.1 (Figure 4). These results revealed that the pcIL-34 and pcHrp1 were successfully expressed in vitro.

### 3.3. Detection of pcIL-34 and pcHrp1 in the Muscle of Immnunized Fish by PCR

**Figure 5:**
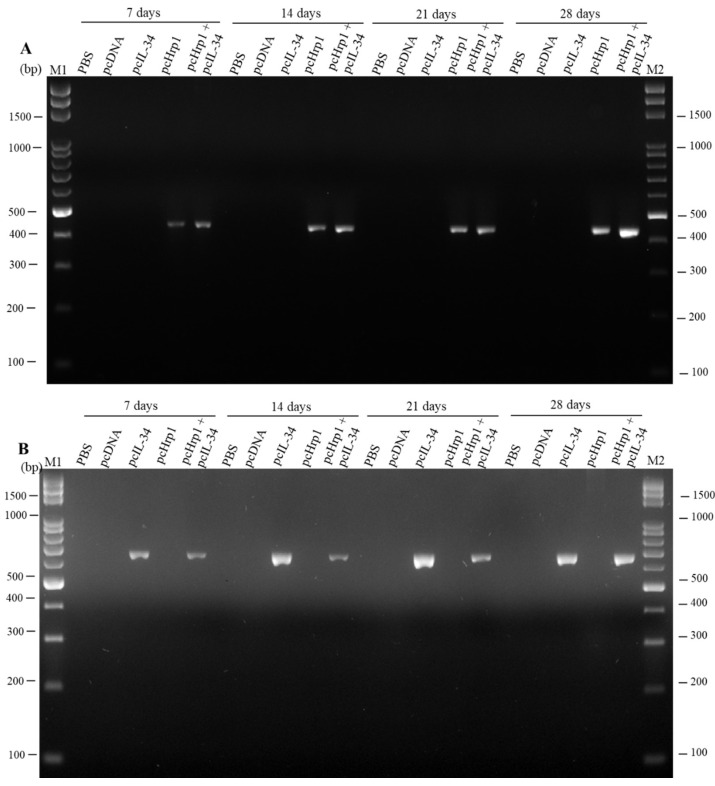
PCR detection of plasmid pcHrp1 and pcIL-34 in muscle tissue of vaccinated fish. (**A**) PCR detection of plasmid DNA pcHrp1 in muscle tissue of vaccinated fish. (**B**) PCR detection of plasmid DNA pcIL-34 in muscle tissue of vaccinated fish. Largemouth bass were injected with phosphate-buffered saline (PBS), or vaccinated with pcDNA3.1, pcIL-34, pcHrp1 and pcHrp1+pcIL-34, respectively. Muscle tissue samples were taken and used for DNA extraction from the vaccinated fish at 7 days, 14 days, 21 days, and 28 days. Lane M1 and M2: 100 bp DNA ladder.

PCR was conducted with the specific primers mentioned above to validate the presence of pcHrp1 and pcIL-34 in the muscle of immunized fish at various time points. A 669 bp PCR product was successfully amplified from extracted DNA of muscle tissue at 7 days, 14 days, 21 days and 28 days p.v. in pcIL-34 and pcHrp1+pcIL-34 groups, but not in PBS, pcDNA3.1, and pcHrp1 groups (Figure 5A). Simultaneously, a PCR product of 435 bp in length was also detected in the pcHrp1 and pcHrp1+pcIL-34 groups; however, no specific band corresponding to the Hrp1 gene was observed in the extracted DNA samples from muscle samples of PBS, pcDNA3.1, and pcIL-34 immunized fish (Figure 5B).

### 3.4. In vivo Transcription Analysis of pcIL-34 and pcHrp1 by RT-PCR

**Figure 6:**
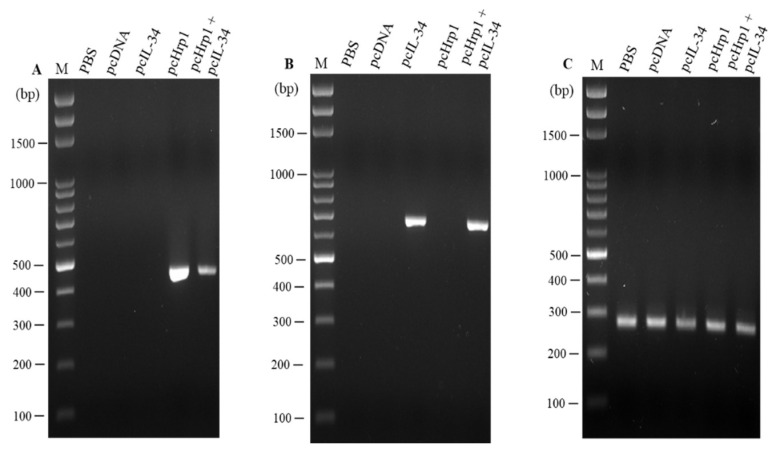
RT-PCR analysis of the Hrp1 gene and exogenous IL-34 gene in the muscle tissue of vaccinated fish 21 days post-vaccination. (**A**) Hrp1 gene; (**B**) exogenous IL-34 gene; (**C**) Rps40 reference gene. M: 100 bp DNA.

On day 21 p.v., transcriptional analysis of the recombinant plasmid pcIL-34 and pcHrp1, in vaccinated largemouth bass muscle tissue, was conducted by RT-PCR. The results showed that the transcription of the IL-34 gene was observed in the muscle tissue of pcIL-34 and pcHrp1+pcIL-34 injected groups; in contrast, no transcription was detected in the PBS, pcDNA3.1 control groups and the pcHrp1 vaccinated group (Figure 6A). Moreover, the transcription of the Hrp1 gene was observed in the muscle tissue of fish vaccinated with pcHrp1 and pcHrp1+pcIL-34, but not in PBS, pcDNA3.1, and pcIL-34 immunized fish (Figure 6B). Control Rps40 gene transcripts were observed in all RNA samples (Figure 6C).

### 3.5. In vivo Expression Analysis of pcIL-34 and pcHrp1 by Western Blotting

**Figure 7:**
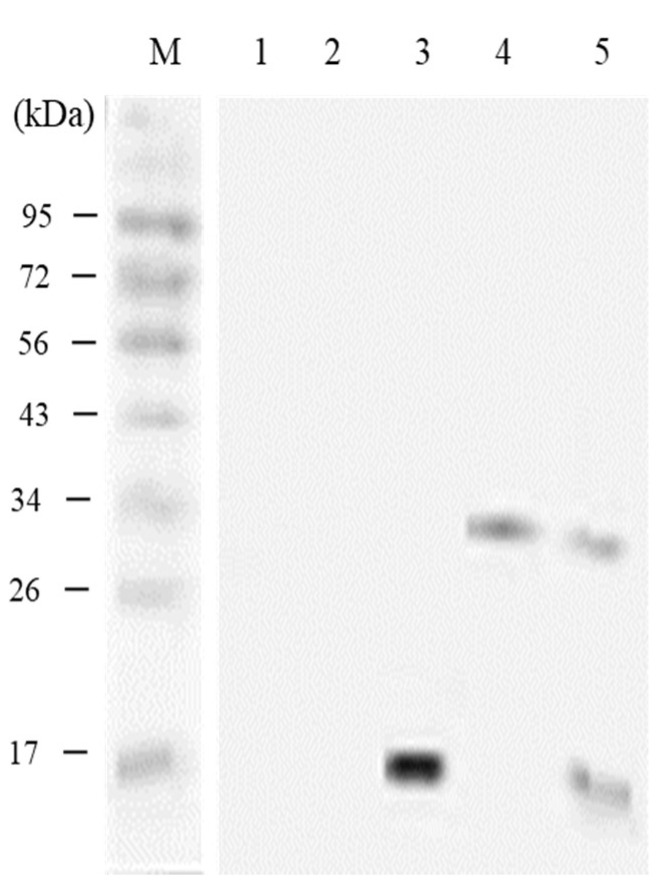
Western blot analysis of the transcription of pcHrp1 and pcIL-34 in immunized fish muscle tissue. M: protein ladder; lanes 1–5: muscle tissue of fish injected with PBS, pcDNA3.1, pcHrp1, pcIL-34 and pcHrp1+pcIL-34, respectively.

The Western blot analysis was conducted to evaluate the expression of exogenous IL-34 and Hrp1 at the translational level in the immunized fish using an anti-histidine antibody. The result demonstrated that specific bands, approximately 15 kDa and 30 kDa, corresponding to expression of the IL-34 and Hrp1 protein, were observed 21 days p.v. at the injection sites of muscle tissues from largemouth bass vaccinated with pcHrp1 and pcIL-34, respectively. Simultaneously, a dual-band with the expected molecular weight of Hrp1 and IL-34 proteins was also detected in the muscle tissues of fish injected with pcHrp1+pcIL-34. However, no bands were observed in fish injected with PBS and pcDNA3.1 (Figure 7). The results indicated that the IL-34 and Hrp1 genes were successfully expressed in the fish.

### 3.6. Serum Lysozyme Activity

**Figure 8:**
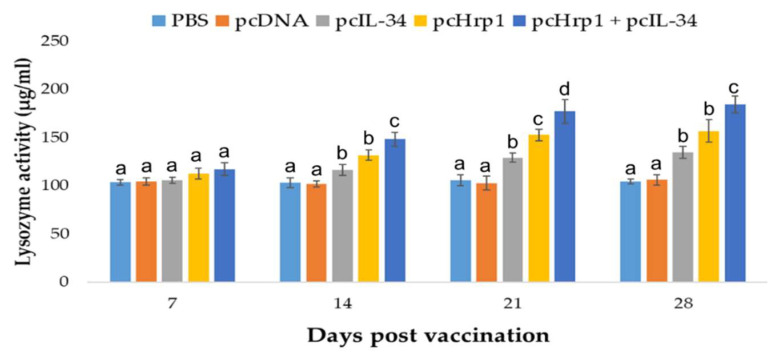
Serum lysozyme activity of vaccinated fish. Largemouth bass were vaccinated with PBS, pcDNA3.1, pcIL-34, pcHrp1 and pcHrp1+pcIL-34, respectively. Sera were collected from 7 days to 28 days post-vaccination. Data are presented as means ± standard deviation (SD) (n = 5). ^a,b,c,d^ A significant difference (*p* < 0.05) exists between groups at the same time point with different superscript letters.

The serum lysozyme activity of immunized and control fish was evaluated utilizing turbidimetry at 7 days, 14 days, 21 days and 28 days p.v. (Figure 8). The groups vaccinated with pcIL-34, pcHrp1, and pcHrp1+pcIL-34 demonstrated significantly higher levels of serum lysozyme activity when compared to those treated with PBS and pcDNA3.1 at 14 days, 21 days and 28 days p.v. No significant difference was recorded between the immunized and control groups at seven days p.v. Moreover, fish vaccinated with pcHrp1 and pcHrp1+pcIL-34 generated remarkably higher serum lysozyme levels when compared with those treated with pcIL-34. The highest lysozyme activity was recorded 28 days p.v. in the pcHrp1+pcIL-34 immunized group (184 µg/mL).

### 3.7. Specific Serum Antibody Production

**Figure 9:**
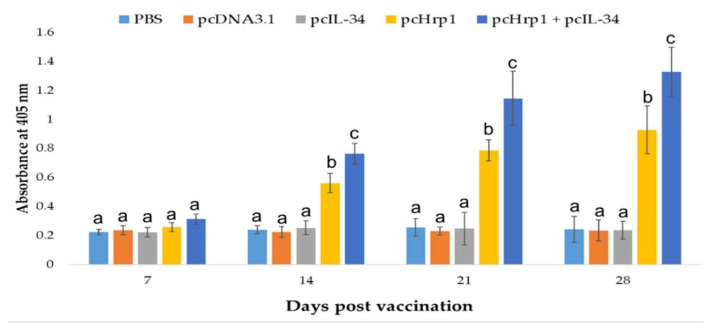
The detection of specific antibody production in immunized fish using ELISA. Largemouth bass were immunized with PBS, pcDNA3.1, pcIL-34, pcHrp1 and pcHrp1+pcIL-34, respectively. Sera were collected from 7 days to 28 days post-vaccination. Data are presented as means ± SD (n = 5). ^a,b,c,d^ A significant difference (*p* < 0.05) exists between groups at the same time point with different superscript letters.

In this experiment, the specific antibody titers in the sera of all experimental fish were measured by Enzyme-linked Immunosorbent Assay (ELISA) on different p.v. days using rHrp1 as the coating antigen (Figure 9). ELISA results demonstrated that no specific anti-rHrp1 antibody was found in fish immunized with PBS, pcDNA3.1, or pcIL-34 at all examined time points. However, fish vaccinated with pcHrp1 and pcHrp1+pcIL-34 generated a significantly higher specific anti-rHrp1 antibody from days 14 to 28 p.v. when compared with the other three groups. Furthermore, the pcHrp1+pcIL-34 group generated significantly higher antibody levels (*p* < 0.05) than pcHrp1 alone at 14 days, 21 days and 28 days p.v.

### 3.8. Expression of Immune-Related Genes

**Figure 10:**
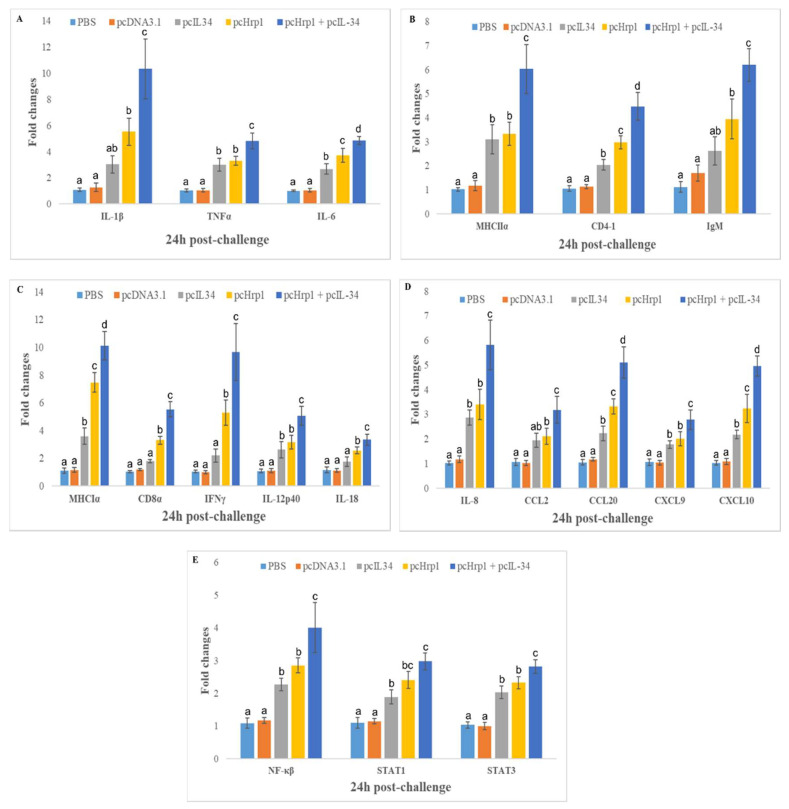
mRNA expression levels of immune-related genes in vaccinated and control fish at 24 h post *N. seriolae* challenge using qRT-PCR. (**A**) Pro-inflammatory cytokines (IL-1β, TNFα, IL-6); (**B**) humoral immunity mediators (MHCIIα, CD4-1, IgM); (**C**) cellular immunity mediators (MHCIα, CD8α, IFNγ, IL-12p40, IL-18); (**D**) chemokines (IL-8, CCL2, CCL20, CXCL9, CXCL10); (**E**) transcriptional factors (NF-κB, STAT1, STAT3). Data are presented as means ± SD (n = 8). ^a,b,c,d^ A significant difference (*p* < 0.05) exists between groups with different superscript letters.

To validate the effect of plasmid DNA immunization on the expression of the immune-related genes listed above, qRT-PCR was performed to confirm their expression levels in the head kidney of fish 24 h post-challenge, using the housekeeping gene Rsp40 and ß-actin as internal controls. The relative expression analysis (Figure 10A–E) indicated that the mRNA expression levels of all investigated genes were significantly induced in the head kidney of the pcHrp1 and pcHrp1+pcIL-34 groups when compared to the expression in the PBS and pcDNA3.1 control groups (*p* < 0.05). Moreover, the co-injection of pcHrp1+pcIL34 could significantly enhance the expression levels of investigated genes when compared to pcHrp1 (except STAT1) and pcIL-34 alone (*p* < 0.05). Fish immunized with pcHrp1+pcIL34 exhibited significantly higher expression levels of IL-1β, MHCI, and IFNγ by more than 10-fold; the expression of IL-8, IL-6, CD8α, IL-12, CCL20, and CXCL10 was increased by more than 5-fold when compared with the PBS and pcDNA3.1 control groups (*p* < 0.05). Furthermore, the qRT-PCR results also indicated that, compared to control groups 24 h post-challenge, the head kidney of fish in the pcIL-34 immunization groups demonstrated notably upregulated TNFα, IL-8, IL-6, MHCI, MHCII (more than 3-fold expression), CD4-1, IL-12, CCL20, CXCL10, STAT1, STAT3, and NF-κB (more than 2-fold expression). Immunization with pcIL-34 also resulted in the upregulation of mRNA levels of CD8α, IL-18, IFNγ, and CCL2; however, it failed to reach statistical difference when compared with PBS and pcDNA3.1 control groups.

### 3.9. Immuno-Protective Efficacy against N. seriolae Infection

**Figure 11:**
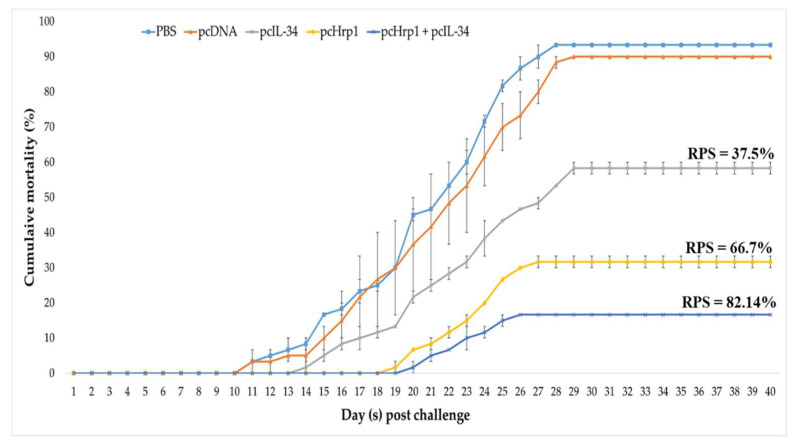
The cumulative mortality of largemouth bass challenged with *N. seriolae*. Each treatment group contained 30 fish. The fish were observed daily for 40 days to calculate the cumulative mortality, and the experiment was conducted in duplicate. Results are presented as means ± SD (n = 2). RPS = relative percent of mortality.

In all experimental groups, the fish survived and revealed no differences in behavioral and pathological signs from that of control fish during the 28-day immunization period. Following the challenge with a virulent strain of *N. seriolae*, the fish in the PBS and pcDNA3.1 control group started dying 11 days post-infection (dpi). The mortality of fish in the control groups persisted until 29 dpi and then stabilized at 30–40 dpi, with no more dead fish recorded. At the end of the experiment, the average cumulative mortality rates in the PBS, pcDNA3.1, pcIL-34, pcHrp1, and pcHrp1+pcIL-34 groups were 93.33%, 90.00%, 58.33%, 31.67%, and 16.67%, respectively, which correlated to an RPS of 3.57% for pcDNA3.1, 37.5% for pcIL-34, 66.07% for pcHrp1, and 82.14% for pcHrp1+pcIL-34 groups when compared with the PBS control group (Figure 11). The dead and moribund fish showed typical signs of nocardiosis, including skin ulcers, anorexia, and multiple white nodular structures in various organs (gills, head kidney, spleen, heart, and mesentery). Pure colonies of *N. seriolae* were re-isolated from the internal organs (head kidney, spleen, liver, and mesentery) of moribund and dead fish during the challenge experiment, confirming that the *N. seriolae* challenge was the only cause of mortality in the experimental fish (Figure S1). At the end of experiments, survival fish from experimental groups were sacrificed to confirm the pathological signs of nocardiosis. Survival fish from the pcIL-34, pcHrp1 and pcHrp1 + pcIL-34 groups did not exhibit any white nodules in internal organs (Figures S2–S4), however, white nodules were found in the liver and mesentery of PBS and pcDNA3.1 survival fish (Figures S5 and S6). These data suggested that pcIL-34 could be used as an effective adjuvant in DNA vaccine development against nocardiosis.

## 4. Discussion

Recently, DNA vaccines have been effective in controlling virus infections and conferring protection against fish bacterial diseases, such as edwardsiellosis, streptococcosis, and vibriosis [56]. Nocardiosis is a widespread disease that can affect various fish species. Hence, the identification and screening for protective antigens from *N. seriolae* can provide an effective candidate for vaccine strategies against this bacterial infection. Hrp1 is a promising antigen with potential in vaccine development against *Mycobacterium tuberculosis* in humans [57]. Reportedly, Hrp1 of *M. tuberculosis* can induce pro-inflammatory responses of the host by modulating the expression of (inducible Nitric Oxide Synthase) iNOS, IL-12, and TNFα [58]; furthermore, it could trigger a protective T-helper type 1 (Th1) response of the host and play a role against *M. tuberculosis* infection [59]. In our previous study, the emulsified recombinant protein HRP1 (Hypoxic response protein 1) of *N. seriolae* with a commercial adjuvant Montanide^TM^ ISA 763 A VG (Seppic, Paris, France) could activate both humoral and cellular mediated immune responses, protecting against nocardiosis in largemouth bass [43]. Therefore, Hrp1 is expected to be a suitable DNA vaccine candidate.

An ideal vaccine candidate mainly comprises of a strong immunogenic antigen and a suitable adjuvant that can help induce innate immunity and generate long-lasting adaptive immune responses. However, most traditional adjuvants, such as aluminum salt, mineral oil (Freund incomplete adjuvant (FIA) or Freund complete adjuvant (FCA) and non-mineral oil (ISA), can sometimes induce undesirable effects, including tissue adhesion and granulomatous lesions at the injection site [21,60,61,62,63]. Hence, the co-administration of antigens with cytokines is a promising strategy to reduce the side effects of adjuvants, as well as to enhance humoral and cell-mediated immune responses for promoting vaccine efficacy. It has been documented that IL-34 has beneficial effects in viral and bacterial infections. In amphibians, IL-34-derived macrophages displayed effective in vitro antiviral activity by generating vigorous gene expression of Arginase 1 (Arg-1), Reactive oxygen species (ROS), Nicotinamide Adenine Dinucleotide Phosphate Oxidase (NADPH-oxidase), and type I interferon gamma against ranavirus FV-3 (Frog Virus 3), and significantly elongated survival of FV-3 infected tadpoles. Reportedly, treatment with IL-34 also assisted to protect mice against polymicrobial sepsis by inducing the expression of cytokines (IL-6, TNFα) and chemokines (CXCL1, CCL2) [54]. In teleost species, IL-34 is reported to be involved in viral, bacterial and parasite infections, such as infection of grass carp (*Ctenopharyngodon idella*) with *Flavobacterium columnare* and spring viremia of carp virus [35], *Vibrio anguillarum* infection in large yellow croaker *(Larimichthys crocea*), parasitic infection of gilthead sea bream (*Sparus aurata*) with *Enteromyxum leei*, and grouper (*Epinephelus coioides*) infected with *Cryptocaryon irritans* [54]. Collectively, IL-34 may become an ideal adjuvant to ameliorate the host immune response against viral and bacterial infection in teleost species.

In the present work, an IL-34 homolog was identified in largemouth bass and described as a potential adjuvant for a DNA vaccine against *N. seriolae* infection. The C-terminus of LMB-IL-34 and other teleost IL-34 proteins possess a conserved cationic lysine/arginine-rich motif (RKG[R/K]K), which does not exist in mammalian IL-34s [32,33,34,35]. It is reported that a similar C-terminal cationic motif (KRKR) of human and fish IFNγ molecules, a nuclear translocation site (NLS), is essential to maintain the biological functions of IFNγ [64]. However, the exact function of the IL-34 cationic motif, located in the C-terminus in teleost species, is yet to be elucidated. In mammals, IL-34 is a secreted glycoprotein which forms homodimers or heterodimers with colony-stimulating factor 1 (CSF1) to act as a macrophage modulator and exert osteoclast and monocyte functions [31]. The multiple potential a.a. residues (Pro-Ser-Thr) responsible for O-glycosylation at the C-terminus of the human IL-34 have been predicted; however, this region is missing in teleost homologs. It is reported that glycosylation could result in a higher protein molecular weight than the predicted size when examined via SDS-PAGE gel. Similar to observations in grass carp IL-34 expressed in Human Embryonic kidney 293T (HEK293T) cells [35], in this study, LMB-IL-34 expressed in E11 cells (about 30 kDa) also differed by approximately 4.6 kDa from the predicted molecular weight (25.39 kDa). The sequence analysis revealed that three N-glycosylation sites were predicted at the N-terminus of LMB-IL-34; hence, the higher molecular weight could be responsible for the N-glycosylation sites of LMB-IL-34.

In this work, we constructed the recombinant plasmids pcHrp1 and pcIL-34. To improve the transcription and translation efficiency and facilitate the detection of recombinant plasmids in vivo and in vitro, a Kozak consensus and 6 × histidine sequences were inserted into the forward and reverse primers, respectively, for PCR amplification of the Hrp1 and IL-34 genes. To confirm the in vitro expression of the plasmid DNA, a Western blot was conducted using lysate from E11 cells transfected with pcIL-34 and pcHrp1. The results indicated that the transfected E11 cell line with recombinant plasmids was able to produce IL-34 and Hrp1 proteins in vitro.

In general, after intramuscular injection, plasmid DNA is subjected to many fates, including uptake by local cells at the injection site, and degradation or redistribution to other tissues [65]. To verify the presence and transcription of the recombinant DNA plasmids in vivo, DNA and RNA from muscle tissues were extracted and subjected to PCR and RT-PCR, respectively. Western blot analysis was performed to confirm the expression at the translational level of pcHrp1 and pcIL-34 in the muscle tissue of immunized fish. The results of PCR and RT-PCR demonstrated that the Hrp1 and IL-34 genes were only found in the muscle tissue of fish immunized with pcHrp1, pcIL-34 or pcHrp1+pcIL-34, indicating that the recombinant DNA plasmids could avoid local degradation and uptake by the muscle cells at the injection site, resulting in expression at transcription levels. Our results are in agreement with studies investigated in other fish species, including channel catfish [23], orange-spotted grouper [47], snakehead fish [16,66], and the Japanese flounder [11,67]. It is reported that cell membrane permeability and susceptibility of foreign DNA to host enzymes is one of the pivotal factors to evaluate the efficiency of the cellular uptake of DNA vaccines [68]; hence, further studies are required to estimate the efficiency of the cellular uptake of pcIL-34, pcHrp1 and pcHrp1+pcIL-34. Consistent with these results, the corresponding bands of IL-34 and Hrp1 proteins were also detected in the muscles of vaccinated fish by Western blot, suggesting that the IL-34 and Hrp1 proteins were successfully expressed in the vaccinated groups, while no bands were observed in the muscle of the PBS and pcDNA3.1 control groups.

Innate immunity constitutes the first line and an important component of the host’s defense against infection. Lysozyme plays a pivotal role in innate immunity due to its ability to lyse Gram-positive bacterial cells via the targeted hydrolysis of the bacterial cell wall peptidoglycan [69]. Lysozymes cleave the β(1→4) glycosidic linkage between N-acetylmuramic acid and *N*-acetyl-d-glucosamine residues in the peptidoglycans of Gram-positive bacterial cell walls, causing lysis of bacterial cell walls, and preventing an invasion of the bacterium [70]. Fish lysozyme is an enzyme with antibiotic properties and has been considered as an indicator of non-specific immune functions against infections. Lysozyme can be found on the body surface, skin, serum, gills, and intestinal tract of teleost species and is considered as a protective factor against bacterial infection due to its antibacterial proprieties [71]. In this work, largemouth bass immunized with pcIL-34, pcHrp1 and pcHrp1+pcIL-34 induced higher serum lysozyme activity 14 days p.v. The augmentation of serum lysozyme activity was observed in vaccinated fish with pcIL-34, pcHrp1, and pcHrp1+pcIL-34, but not in PBS and pcDNA3.1(+), suggesting that lysozymes may be involved in the innate immune response and play a role in the elimination of *N. seriolae* at an early stage of infection in largemouth bass. Furthermore, the vaccination of fish with certain antigens results in an augmentation of antibodies and serum lysozyme production, with the increase in antibody production being directly proportional to the serum lysozyme activity [71]. In line with the serum lysozyme titers, the anti-rHRP1 specific antibody titers in pcHrp1 and pcHrp1+pcIL34 immunized groups were significantly induced from day 14 p.v., and persisted up to 21 days and 28 days p.v. when compared with the other groups, including PBS, pcDNA3.1(+), and pcIL34. The results of serum lysozyme activity and specific antibody titers indicated that pcHrp1 and pcHrp1+pcIL-34 could induce innate and adaptive humoral immune responses in vaccinated largemouth bass, and thus might contribute to the protection against nocardial infection.

Specific antibodies have demonstrated effective protection against certain intracellular pathogens, including *Edwardsiella tarda* [72], *M. tuberculosis* [73], *Burkholderia pseudomallei* [74], *Francisella tularensis* [75], and *Yersinia enterocolitica* [76]. However, this might not adequate for effective protection, requiring the participation of humoral and cell-mediated immune responses of the host. Therefore, the qRT-PCR analysis of immune-related genes in the head kidney of vaccinated fish at 24 h post-challenge was performed. The results indicated that largemouth bass immunized with pcHrp1 or pcHrp1+pcIL-34 could significantly increase the mRNA expression levels of immune-related genes responsible for inflammatory responses (IL-1β, TNF-α, and IL-6), cell-mediated immunity (MHCIα, CD8α, IL-12p40, IL-18, and IFNγ), humoral immunity (MHCIIα, CD4-1, and IgM), and chemokines (IL-8, CCL2, CCL20, CXCL9, and CXCL10). The upregulation of MHCIα, MHCIIα, CD8α, and CD4-1 suggested that the specific cell-mediated immune responses, especially the CD8 cytotoxic T lymphocyte and CD4+ Th cell responses, were elicited in pcHrp1 or pcHrp1+pcIL-34 vaccinated fish. The transcriptional expression results also demonstrated that the mRNA expression levels of all investigated genes in pcHrp1+pcIL-34 vaccinated fish were remarkably upregulated when compared with pcHrp1 immunization alone.

Chemokines are important molecules with different functions, including inflammatory mediators, immuno-modulators, lymphopoiesis, and antimicrobial activities [77]. The early induction of chemokines during intracellular bacteria invasion is an important step for the recruitment of antigen-presenting cells (APC) and normal functioning of phagocytes; furthermore, they act as the first barrier against pathogens [78]. Advances in the understanding of chemokine functions during infection have demonstrated that inducible chemokines are involved in both innate and adaptive immune responses [77,79]. Reportedly, chemokines are specialized in the recruitment of various cells, including neutrophils (IL-8), monocytes (CCL2), Th1 cells (CXCL9, CXCL10), Th2 cells (CCL17, CCL21), and Th17 cells (CCL20), to sites of infection. Additionally, several chemokines possess antimicrobial activities, including CXCL9, CXCL10, CXCL11, CXCL6, CXCL14, CCL2, CCL20, and CCL28 [79,80], which could be beneficial in controlling certain intracellular microorganisms, including *Salmonella enteritica* [81], *Neisseria gonorrhoeae* [82], *Listeria monocytogenes* [83], *Leishmania Mexicana* [84], *Plasmodium falciparum* [85], and *Mycobacterium tuberculosis* [86]. Our results demonstrated that vaccination with pcHrp1 or pcHrp1+pcIL-34 could significantly trigger the expression of IL-8, CCL2, CXCL9, CXCL10, and CCL20, indicating that the chemokines were involved in the recruitment of neutrophils, monocytes, Th1 cells, and Th17 cells to the infection sites. Furthermore, the antimicrobial activities of CXCL9, CXL10, CCL2, and CCL20 could contribute to the host’s defense against nocardiosis infection.

Moreover, vaccination with pcIL-34 alone could activate different molecular signaling pathways, including NF-κB, STAT1, and STAT3 transcriptional factors, to promote innate and adaptive immune responses by augmenting the expression of pro-inflammatory cytokines (IL-6, IL12p40, TNFα), Th1 related chemokines (CXCL9, CXCL10), and Th17 related chemokines (CCL20). These results are similar to previous studies on mammalian IL-34 [87,88,89]. The above data indicated that the pcHrp1 vaccine could trigger innate and adaptive immune responses, while pcIL-34 immunization as an adjuvant could induce inflammatory responses and enhance vaccine efficacy against *N. seriolae* infections in largemouth bass. In this work, the low immune responses of pcIL-34 compared to pcHrp1 might be due to the affinity for major histocompatibility complex-encoded molecules (MHCI and MHCII). This affinity may affect the character and the potency of the response. Furthermore, secreted IL-34 is reported to bind to the extracellular domain of colony-stimulating factor 1 receptor (CSF-1R), resulting in activation of several signaling pathways that modulate major cellular functions, including cytokine and chemokine expression [54]. Hence, the exact mechanism of pcIL-34 in stimulating immune responses in fish requires further investigation, as this is the first report on the adjuvanticity of teleost IL-34.

In the present study, largemouth bass were vaccinated with 25 μg of plasmid DNA, the same dose of plasmid DNA was applied in various studies of DNA vaccine development against viral and bacterial diseases in fish such as infectious pancreatic necrosis virus [90], spring viremia of carp virus [91], red seabream iridovirus [92], *Mycobacterium marinum* [93], and *N. seriolae* infection [66,94]. In our previous study, largemouth bass immunized with 10 μg of plasmid DNA encoding Resuscitation promoting factor B (RpfB) and Low molecular weight T-cell antigen (TB8.4) could not provide effective protection with an RPS value of 6.25% and 39% respectively. However, immunization of largemouth bass with 25 μg of pcTB8.4 could generate better protection with an RPS = 51.7%.

One approach to evaluate the efficacy of fish vaccines is to determine the RPS of vaccinated fish when compared to control fish after a lethal challenge with virulent pathogens [95]. In the present work, largemouth bass vaccinated with pcIL-34, pcHrp1, and pcHrp1+pcIL-34 were protected against *N. seriolae* infection with an RPS value of 37.5%, 66.07%, and 82.14%, respectively. As expected, the co-injection of pcIL-34 with pcHrp1 not only strengthens the humoral and cell-mediated immune responses, but also enhances the protective efficacy against nocardiosis in largemouth bass. In our previous study, we have demonstrated that the recombinant protein rHRP1 emulsified with commercial Montanide^TM^ ISA 763 A VG adjuvant triggers both humoral and cellular immune responses in immunized largemouth bass, conferring an RPS of 73.33% [38]. Considering the immune protection provided by rHRP1 emulsified with ISA 763 A VG, the recombinant plasmid DNA pcHrp1 evaluated in this study may confer less protection as it resulted in a lower RPS (66.07%). The protection afforded by both the recombinant protein vaccine and DNA vaccine, based on Hrp1, might primarily involve the antigen’s capacity to induce humoral and cell-mediated immune responses. However, various factors, such as vaccine type, antigen dose, vaccination schedule (with or without booster), vaccine delivery route and the application of adjuvants can influence the vaccine efficacy [95]. Similarly, recent vaccine studies in *Vibrio alginolyticus* [47,96], *Vibrio anguillarum* [10,11,97,98], *Vibrio harveyi* [48], and *Edwardsiella tarda* [13] indicated that vaccination of recombinant proteins in combination with adjuvants could confer higher RPS levels than immunization with a DNA vaccine alone. Interestingly, pcHrp1, when co-administrated with pcIL-34, could enhance the protection by generating better RPS (82.14%) compared with rHRP1 emulsified with ISA 763 A VG; hence, this result might explain the superiority of IL-34 as an molecular adjuvant. Therefore, the combination of a strong immunogenic antigen and a suitable molecular adjuvant that can induce the innate and adaptive immune responses of the host against invading pathogens is the ideal way to augment DNA vaccine efficacy.

## 5. Conclusions

Collectively, our present results indicated that pcIL-34 served as a novel molecular adjuvant when co-injected with the DNA vaccine pcHrp1 to enhance protection and trigger both innate and adaptive immunity (humoral and cell-mediated responses) of largemouth bass against *N. seriolae* infection. Therefore, interleukin-34 can be used as a promising adjuvant in DNA vaccines to prevent bacterial infection in fish. To the best of our knowledge, this is the first study evaluating the adjuvanticity of IL-34 in a DNA vaccination model in fish.

## Figures and Tables

**Table 1:** Primers used in this study.

Primer Name	Primer Sequence (5′ → 3′)	Application	References
Hrp1-F	TTGCTAGC**GCCACCATG**GCCACGGCACG (*NheI*)	Plasmid construction, PCR, and RT-PCR	This study
Hrp1-R	GTAAGCTTCTA**ATGATGATGATGATGATG**TGTCCAAGGCGCGCA (*HindIII*)	Plasmid construction	
Hrp1-R2	CTATGTCCA AGGCGCGCAGACC	PCR and RT-PCR	
IL-34-F	**CG**AAGCTT**GCCACCATG**GTTCTACTGTGCA (*HindIII*)	Plasmid construction, PCR, and RT-PCR	
IL-34-R	GAATTCTCA**ATGATGATGATGATGATG**GCTTTCGGGCTGTAA *(EcoRI)*	Plasmid construction	
IL-34-R2	TCAGCTTTCGGGCTGTAAGTCTTTC	PCR and RT-PCR	
RPS40	F: CAGAAATGGCACGATAAGCA	qRT-PCR	Martyniuk et al., 2016
	R: GACCTTTACGCCCAAATCC		
β-actin	F: CCACCACAGCCGAGAGGGAA	qRT-PCR	Omkar et al, 2016
	R: TCATGGTGGATGGGGCCAGG		
IL-1β	F: TTGCCATAGAGAGGTTTA	qRT-PCR	
	R: ACACTATATGCTCTTCCA		
IL-12p40	F: TCTTCCATCCTTGTGGTCTTCC	qRT-PCR	
	R: CAGTTCCAGGTCAAAGTGGTC		
TNFα	F: CTAGTGAAGAACCAGATTGT	qRT-PCR	
	R: AGGAGACTCTGAACGATG		
IL-8	F: GAGCCATTTTTCCTGGTGACT	qRT-PCR	
	R: TCCTCATTGGTGCTGAAAGATC		
NF-κB	F: AGGATGACTGAAGCTCCGTT	qRT-PCR	
	R: GGACACGAGGAGGATCGGAGT		
IFNγ	F: TGCAGGCTCTCAAACACATC	qRT-PCR	Hoang et al, 2020
	R: TGTTTTCGGTCAGTGTGCTC		
MHCI-α	F: GTGGTTCAACGTCAACATCG	qRT-PCR	
	R: ACCCAGACTTGTTCGGTGTC		
MHCII-α	F: GAGGACCTTGCTGTCATTGG	qRT-PCR	
	R: GCGTACCAAACCTCTTCACC		
CD4-1	F: GCTCCAGCGGGGAATAATTT	qRT-PCR	
	R: GCCAGGCAAGCTCAAAGTTA		
CD8-α	F: GGAAGGGGATCCTGTTGACA	qRT-PCR	
	R: CCAGCACTCGAAACCAGATG		
IgM	F: CTGGACCAGTCTCCCTCTGA	qRT-PCR	
	R: CGAGGTACTGAGTGCTGCTG		
STAT3	F: CCACCCAAAGAACGTGAACT	qRT-PCR	
	R: TCAATGGTCAGGCCTCTCTT		
CCL20	F: ACAACCACGGAAAACTGCCG	qRT-PCR	
	R: TCCTCACCCACTCATCCTTC		
STAT1	F: TAAAACTCCGGTTCCTGGTG	qRT-PCR	This study
	R: CCGTTTGACTCCTCCATGTT		
IL-18	F: TTGATGGCAAGAAGATGGTGG	qRT-PCR	
	R: AAGCCTTGTGTGCAGTTTCCT		
IL-6	F: GGAACCCTGAACAGGTAACG	qRT-PCR	
	R: TGTGCGGTCATCTTTCTGTGG		
CCL2	F: GCGAGTGGTCAGCTACATCA	qRT-PCR	
	R: GATGAGCTCCTTCACCCAAG		
CXCL9	F: GGAAGATGTTTGTGTCCACAG	qRT-PCR	
	R: GGCGTTTTGGGTAGACTGTG		
CXCL10	F: GAATCGGGACAGCAGTGTCT	qRT-PCR	
	R: CAGTTGCTGGGTAGATCTGGA		

Hrp1 = hypoxic response protein 1; IL = interleukin; TNF = tumor necrosis factor; NF-κB = nuclear factor-kappa light chain enhancer of activated B cells; IFN = interferon; MHC = major histocompatibility complex; CD = cluster of differentiation; Ig = immunoglobulin; STAT = signal transducer and activator of transcription; CCL = chemokine (C-C motif) ligand; CXCL = chemokine (C-X-C motif) ligand. Underlined nucleotides are restriction sites of the enzyme indicated in the brackets, and nucleotides in bold are Kozak consensus and 6× histidine tag sequences, respectively.

## Supplementary Materials

The following are available online at https://www.mdpi.com/2076-393X/8/2/151/s1, Figure S1. Multiple white nodules found in different organs of dead fish and re-isolation of N. seriolae. (A) Head kidney; (B) Liver; (C) Mesentery; (D) Spleen; (E) Pure colonies of N. seriolae growth in BHI-agar. Red arrow indicated the white nodules, Figure S2. Pathological signs of survival fish in pcIL-34 vaccinated group. (A) Side view of fish; (B) No ulcer found in ventral side of fish – injection site (yellow arrows); (C) Liver; (D) Mesentery, Figure S3. Pathological signs of survival fish in pcHrp1 vaccinated group. (A) Side view of fish; (B) No ulcer found in ventral side of fish – injection site (yellow arrows); (C) Liver; (D) Mesentery, Figure S4. Pathological signs of survival fish in pcHrp1 + pcIL-34 vaccinated group. (A) Side view of fish; (B) No ulcer found in ventral side of fish – injection site (yellow arrows); (C) Liver; (D) Mesentery, Figure S5. Pathological signs of survival fish in PBS injected group. (A) Side view of fish with ulcer at injection site (yellow arrows); (B) Ulcer found in ventral side of fish – injection site (yellow arrows); (C) Nodules found in liver (red arrow); (D) Nodule found in mesentery (red arrow), Figure S6. Pathological signs of survival fish in pcDNA3.1 injected group. (A) Side view of fish with ulcer at injection site (yellow arrows); (B) Ulcer found in ventral side of fish – injection site (yellow arrows); (C) Nodules found in liver (red arrow); (D) Nodule found in mesentery (red arrow).

## References

[1] Kariya T., Kubota S., Nakamura Y., Kira K. (1968). Nocardia infection in cultured yellowtails (*Seriola quinqueradiata* and *S. purpursacens*) I. Bacteriological study. Fish Pathol..

[2] Chen S.C., Lee J.L., Lai C.C., Gu Y.W., Wang C.T., Chang H.Y. (2000). Nocardiosis in sea bass, *Lateolabrax japonicus*, in Taiwan. J. Fish Dis..

[3] Huang S.L., Lai K.C., Su S.C., Shei M.C., Chen S.N. (2004). Isolation and characterization of the pathogenic bacterium, *Nocardia seriolae*, from female broodstock of striped mullet (*Mugil cephalus*). J. Fish. Res..

[4] Labrie L., Ng J., Tan Z., Komar C., Ho E., Grisez L., Reantaso M.G.B., Mohan V., Crumlish M., Subasinghe R.P. (2008). Nocardial infections in fish: An emerging problem in both freshwater and marine aquaculture systems in Asia. Diseases in Asian Aquaculture VI.

[5] Vu-Khac H., Duong V.Q.B., Chen S.C., Pham T.H., Nguyen T.T.G., Trinh T.T.H. (2016). Isolation and genetic characterization of *Nocardia seriolae* from snubnose pompano *Trachinotus blochii* in Vietnam. Dis. Aquat. Org..

[6] Shimahara Y., Huang Y.F., Tsai M.A., Wang P.C., Yoshida T., Lee J.L., Chen S.C. (2009). Genotypic and phenotypic analysis of fish pathogen, Nocardia seriolae, isolated in Taiwan. Aquaculture.

[7] Silveira M.M., Oliveira L., Schuch R.A., McBride A.J.A., Dellagostin O.A., Hartwig D.D. (2017). DNA vaccines against leptospirosis: A literature review. Vaccine.

[8] McNeel D.G., Becker J.T., Johnson L.E., Olson B.M. (2012). DNA Vaccines for Prostate Cancer. Curr. Cancer Ther. Rev..

[9] Liu C., Hu X., Cao Z., Sun Y., Chen X., Zhang Z. (2019). Construction and characterization of a DNA vaccine encoding the SagH against *Streptococcus iniae*. Fish Shellfish Immunol..

[10] Xu H., Xing J., Tang X., Sheng X., Zhan W. (2019). Intramuscular administration of a DNA vaccine encoding OmpK antigen induces humoral and cellular immune responses in flounder (*Paralichthys olivaceus*) and improves protection against *Vibrio anguillarum*. Fish Shellfish Immunol..

[11] Xing J., Xu H., Tang X., Sheng X., Zhan W. (2019). A DNA Vaccine Encoding the VAA Gene of *Vibrio anguillarum* Induces a Protective Immune Response in Flounder. Front. Immunol..

[12] Jiao X.D., Zhang M., Hu Y., Sun L. (2009). Construction and evaluation of DNA vaccines encoding *Edwardsiella tarda* antigens. Vaccine.

[13] Sun Y., Liu C.S., Sun L. (2011). Comparative study of the immune effect of an *Edwardsiella tarda* antigen in two forms: Subunit vaccine vs DNA vaccine. Vaccine.

[14] Liu X., Xu J., Zhang H., Liu Q., Xiao J., Zhang Y. (2016). Design and evaluation of an *Edwardsiella tarda* DNA vaccine co-encoding antigenic and adjuvant peptide. Fish Shellfish Immunol..

[15] Kato G., Kato K., Jirapongpairoj W., Kondo H., Hirono I. (2014). Development of DNA vaccines against *Nocardia seriolae* infection in fish. Fish Pathol..

[16] Chen J., Wang W., Hou S., Fu W., Cai J., Xia L., Lu Y. (2019). Comparison of protective efficacy between two DNA vaccines encoding DnaK and GroEL against fish nocardiosis. Fish Shellfish Immunol..

[17] Salonius K., Simard N., Harland R., Ulmer J.B. (2007). The road to licensure of a DNA vaccine. Curr. Opin. Investig. Drugs.

[18] Dalmo R.A. (2018). DNA vaccines for fish: Review and perspectives on correlates of protection. J. Fish Dis..

[19] Ingolotti M., Kawalekar O., Shedlock D.J., Muthumani K., Weiner D.B. (2010). DNA vaccines for targeting bacterial infections. Expert Rev. Vaccines.

[20] Reynes-Cerpa S., Maisey K., Reyes-López F., Toro-Ascuy D., Sandino A.M., Imarai M. (2012). Fish cytokines and immune response. New Adv. Contrib. Fish Biol..

[21] Tafalla C., Bøgwald J., Dalmo R.A. (2013). Adjuvants and immunostimulants in fish vaccines: Current knowledge and future perspectives. Fish Shellfish Immunol..

[22] Guo M., Tang X., Sheng X., Xing J., Zhan W. (2017). The Immune Adjuvant Effects of Flounder (*Paralichthys olivaceus*) Interleukin-6 on *E. tarda* Subunit Vaccine OmpV. Int. J. Mol. Sci..

[23] Wang E., Long B., Wang K., Wang J., He Y., Wang X., Yang Q., Liu T., Chen D., Geng Y. (2016). Interleukin-8 holds promise to serve as a molecular adjuvant in DNA vaccination model against *Streptococcus iniae* infection in fish. Oncotarget.

[24] Cao Y., Zhang Q., Xu L., Li S., Wang D., Zhao J., Liu H., Feng J., Lu T. (2017). Effects of different cytokines on immune responses of rainbow trout in a virus DNA vaccination model. Oncotarget.

[25] Guo M., Tang X., Sheng X., Xing J., Zhan W. (2018). The effects of IL-1β, IL-8, G-CSF and TNF-α as molecular adjuvant on the immune response to an *E. tarda* subunit vaccine in flounder (*Paralichthys olivaceus*). Fish Shellfish Immunol..

[26] Xu H., Xing J., Tang X., Sheng X., Zhan W. (2019). Generation and functional evaluation of a DNA vaccine co-expressing *Vibrio anguillarum* VAA protein and flounder interleukin-2. Fish Shellfish Immunol..

[27] Wang E., Liu T., Wu J., Wang K., Chen D., Geng Y., Huang X., Ouang P., Lai W., Ai X. (2019). Molecular characterization, phylogenetic analysis and adjuvant effect of channel catfish interleukin-1βs against *Streptococcus iniae*. Fish Shellfish Immunol..

[28] Matsumoto M., Araki K., Hayashi K., Takeuchi Y., Shiozaki K., Suetake H., Yamamoto A. (2017). Adjuvant effect of recombinant interleukin-12 in the Nocardiosis formalin-killed vaccine of the amberjack *Seriola dumerili*. Fish Shellfish Immunol..

[29] Xu H., Xing J., Tang X., Sheng X., Zhan W. (2019). The effects of CCL3, CCL4, CCL19 and CCL21 as molecular adjuvants on the immune response to VAA DNA vaccine in flounder (*Paralichthys olivaceus*). Dev. Comp. Immunol..

[30] Kumari R., Kole S., Soman P., Rathore G., Triphathi G., Makesh M., Rajendran K.V., Bedekar M.K. (2018). Bicistronic DNA vaccine against *Edwardsiella tarda* infection in *Labeo rohita*: Construction and comparative evaluation of its protective efficacy against monocistronic DNA vaccine. Aquaculture.

[31] Ge Y., Huang M., Yao Y.M. (2019). Immunomodulation of interleukin-34 and its potential significance as a disease Biomarker and Therapeutic target. Int. J. Biol. Sci..

[32] Wang T., Kono T., Monte M., Kuse H., Costa M., Korenaga H., Maehr T., Husain M., Sakai M., Secombes C.J. (2013). Identification of IL-34 in teleost fish: Differential expression of rainbow trout IL-34, MCSF1 and MCSF2, ligands of the MCSF receptor. Mol. Immunol..

[33] Mo Z.Q., Li Y.W., Zhou L., Li A.X., Luo X.C., Dan X.M. (2015). Grouper (*Epinephelus coioides*) IL-34/MCSF2 and MCSFR1/MCSFR2 were involved in mononuclear phagocytes activation against *Cryptocaryon irritans* infection. Fish Shellfish Immunol..

[34] Wang L., Jiang L., Wu C., Lou B. (2018). Molecular characterization and expression analysis of large yellow croaker (*Larimichthys crocea*) interleukin-12A, 16 and 34 after poly I:C and *Vibrio anguillarum* challenge. Fish Shellfish Immunol..

[35] Xue Y., Jiang X., Gao J., Li X., Xu J., Wang J., Gao Q., Zou J. (2019). Functional characterisation of interleukin 34 in grass carp *Ctenopharyngodon idella*. Fish Shellfish Immunol..

[36] Miyoshi Y., Suzuki S. (2003). A PCR method to detect *Nocardia seriolae* in fish samples. Fish Pathol..

[37] Rombel I.T., Sykes K.F., Rayner S., Johnston S.A. (2002). ORF-FINDER: A vector for high-throughput gene identification. Gene.

[38] Wilkins M.R., Gasteiger E., Bairoch A., Sanchez J.C., Williams K.L., Appel R.D., Hochstrasser D.F. (1999). Protein identification and analysis tools in the ExPASy server. Methods Mol. Biol..

[39] Almagro Armenteros J.J., Tsirigos K.D., Sønderby C.K., Petersen T.N., Winther O., Brunak S., Heijne von G., Nielsen H. (2019). SignalP 5.0 improves signal peptide predictions using deep neural networks. Nat. Biotechnol..

[40] Pitti T., Chen C.T., Lin H.N., Choong W.K., Hsu W.L., Sung T.Y. (2019). N-GlyDE: A two-stage N-linked glycosylation site prediction incorporating gapped dipeptides and pattern-based encoding. Sci. Rep..

[41] Ye J., McGinnis S., Madden T.L. (2006). BLAST: Improvements for better sequence analysis. Nucleic Acids Res..

[42] Kozak M. (1991). Structural features in eukaryotic mRNAs that modulate the initiation of translation. Biol. Chem..

[43] Hoang H.H., Wang P.C., Chen S.C. (2020). The protective efficacy of recombinant hypoxic response protein 1 of *Nocardia seriolae* in largemouth bass (*Micropterus salmoides*). Vaccine.

[44] Nguyen T.T.T., Nguyen H.T., Wang P.C., Chen S.C. (2017). Identification and expression analysis of two pro-inflammatory cytokines, TNF-alpha and IL-8, in cobia (*Rachycentron canadum* L.) in response to *Streptococcus dysgalactiae* infection. Fish Shellfish Immunol..

[45] Byadgi O., Chen C.W., Wang P.C., Tsai M.A., Chen S.C. (2016). De Novo Transcriptome Analysis of Differential Functional Gene Expression in Largemouth Bass (*Micropterus salmoides*) after Challenge with *Nocardia seriolae*. Int. J. Mol. Sci..

[46] Panzarin V., Toffan A., Abbadi M., Buratin A., Mancin M., Braaen S., Olsen C.M., Bargelloni L., Rimstad E., Cattoli G. (2016). Molecular Basis for Antigenic Diversity of Genus Betanodavirus. PLoS ONE.

[47] Huang Y., Cai S., Pang H., Jian J., Wu Z. (2017). Immunogenicity and efficacy of DNA vaccine encoding antigenic AcfA via addition of the molecular adjuvant Myd88 against *Vibrio alginolyticus* in *Epinephelus coioides*. Fish Shellfish Immunol..

[48] Wang Q., Chen J., Liu R., Jia J. (2011). Identification and evaluation of an outer membrane protein OmpU from a pathogenic *Vibrio harveyi* isolate as vaccine candidate in turbot (*Scophthalmus maximus*). Lett. Appl. Microbiol..

[49] Xu L., Zhao J., Liu M., Ren G., Jian F., Yin J., Feng J., Liu H., Lu T. (2017). Bivalent DNA vaccine induces significant immune responses against infectious hematopoietic necrosis virus and infectious pancreatic necrosis virus in rainbow trout. Sci. Rep..

[50] Nguyen H.T., Nguyen T.T.T., Chen Y.C., Vu-Khac H., Wang P.C., Chen S.C. (2018). Enhanced immune responses and effectiveness of refined outer membrane protein vaccines against *Vibrio harveyi* in orange-spotted grouper (*Epinephelus coioides*). J. Fish Dis..

[51] Ellis A.E. (1990). Lysozyme Assays. InTech. Fish Immunol..

[52] Martyniuk C.J., Doperalski N.J., Prucha M.S., Zhang J.L., Kroll K.J., Conrow R., Barber D.S., Denslow N.D. (2016). High contaminant loads in Lake Apopka’s riparian wetland disrupt gene networks involved in reproduction and immune function in largemouth bass. Comp. Biochem. Physiol. D Genom. Proteom..

[53] Livak K.J., Schmittgen T.D. (2001). Analysis of relative gene expression data using real-time quantitative PCR and the 2(-Delta Delta C(T)) method. Methods.

[54] Baghdadi M., Umeyama Y., Hama N., Kobayashi T., Han N., Wada H., Seino K.I. (2018). Interleukin-34, a comprehensive review. J. Leukoc. Biol..

[55] Liu H., Leo C., Chen X., Wong B.R., Williams L.T., Lin H., He X. (2012). The mechanism of shared but distinct CSF-1R signaling by the non-homologous cytokines IL-34 and CSF-1. Biochim. Biophys. Acta.

[56] Hølvold L.B., Myhr A.I., Dalmo R.A. (2014). Strategies and hurdles using DNA vaccines to fish. Vet. Res..

[57] Roupie V., Romano M., Zhang L., Korf H., Lin M.Y., Franken K.L.M.C. (2007). Immunogenicity of eight dormancy regulon-encoded proteins of *Mycobacterium tuberculosis* in DNA-vaccinated and tuberculosis-infected mice. Infect. Immun..

[58] Bashir N., Kounsar F., Mukhopadhyay S., Hasnain S.E. (2010). *Mycobacterium tuberculosis* conserved hypothetical protein rRv2626c modulates macrophage effector functions. Immunology.

[59] Singh S., Sharma M., Chaudhry A., Sharma S. (2019). Rv2626c and Rv2032 activate TH1 response and downregulate regulatory T cells in peripheral blood mononuclear cells of tuberculosis patients. Comp. Immunol. Microbiol. Infect. Dis..

[60] Gjessing M.C., Falk K., Weli S.C., Koppang E.O., Kvellestad A. (2012). A sequential study of incomplete Freund’s adjuvant-induced peritonitis in Atlantic cod. Fish Shellfish Immunol..

[61] Veenstra K.A., Wang T., Alnabulsi A., Douglas A., Russell K.S., Tubbs L., Arous J.B., Secombes C.J. (2017). Analysis of adipose tissue immune gene expression after vaccination of rainbow trout with adjuvanted bacterins reveals an association with side effects. Mol. Immunol..

[62] Xu W., Jiao C., Bao P., Liu Q., Wang P., Zhang R., Liu X., Zhang Y. (2019). Efficacy of Montanide^TM^ ISA 763 A VG as aquatic adjuvant administrated with an inactivated *Vibrio harveyi* vaccine in turbot (*Scophthalmus maximus* L.). Fish Shellfish Immunol..

[63] Sun X., Jin P., Liu Q., Wang Q., Zhang Y., Liu X. (2020). A CpG-riched plasmids as vaccine adjuvant reduce antigen dose of an inactivated *Vibrio anguillarum* vaccine in turbot (*Scophthalamus maximus* L.). Fish Shellfish Immunol..

[64] Zou J., Carrington A., Collet B., Dijkstra J.M., Yoshiura Y., Bols N., Secombes C. (2005). Identification and bioactivities of IFN-γ in rainbow trout *Oncorhynchus mykiss*: The first Th1-type cytokine characterized functionally in fish. J. Immunol..

[65] Tonheim T.C., Bøgwald J., Dalmo R.A. (2008). What happens to the DNA vaccine in fish? A review of current knowledge. Fish Shellfish Immunol..

[66] Chen J., Chen Z., Wang W., Hou S., Cai J., Xia L., Lu Y. (2020). Development of DNA vaccines encoding ribosomal proteins (RplL and RpsA) against *Nocardia seriolae* infection in fish. Fish Shellfish Immunol..

[67] Sun Y., Hu Y.H., Liu C.S., Sun L. (2012). Construction and comparative study of monovalent and multivalent DNA vaccines against *Streptococcus iniae*. Fish Shellfish Immunol..

[68] Lee L.Y.Y., Izzard L., Hurt A.C. (2018). A Review of DNA Vaccines Against Influenza. Front. Immunol..

[69] Ragland S.A., Criss A.K. (2017). From bacterial killing to immune modulation: Recent insights to the function of lysozyme. PLoS Pathog..

[70] Brott A.S., Clarke A.J. (2019). Peptidoglycan O-Acetylation as a Virulence Factor: Its Effect on Lysozyme in the Innate Immune System. Antibiotics (Basel).

[71] Saurabh S., Sahoo P.K. (2008). Lysozyme: An important defence molecule of fish innate immune system. Aquac. Res..

[72] Tang X., Liu F., Sheng X., Xing J., Zhan W. (2018). Recombinant NADP-dependent isocitrate dehydrogenase of *Edwardsiella tarda* induces both Th1 and Th2 type immune responses and evokes protective efficacy against edwardsiellosis. Vaccine.

[73] Prados-Rosales R., Carreño L., Cheng T., Blanc C., Weinrick B., Malek A., Lowary T., Baena A., Joe M., Bai Y. (2017). Enhanced control of *Mycobacterium tuberculosis* extrapulmonary dissermination in mice by an arabinomannan-protein conjugate vaccine. PLoS Pathog..

[74] Scott A.E., Burtnick M.N., Stokes M.G., Whelan A.O., Williamson E.D., Atkins T.P., Prior J.L., Brett P.J. (2014). *Burkholderia pseudomallei* capsular polysaccharide conjugates provide protection against acute melioidosis. Infect. Immun..

[75] Rawool D.B., Bitsaktsis C., Li Y., Gosselin D.R., Lin Y., Kurkure N.V., Metzger D.W., Gosselin E.J. (2008). Utilization of Fc receptors as a mucosal vaccine strategy against an intracellular bacterium, *Francisella tularensis*. J. Immunol..

[76] Singh A.K., Kingston J.J., Murali H.S., Batra H.V. (2014). A recombinant bivalent fusion protein rVE confers active and passive protection against *Yersinia enterocolitica* infection in mice. Vaccine.

[77] Zlotnik A., Yoshie O. (2000). Chemokines: A new classification system and their role in immunity. Immunity.

[78] Ismail N., Olano J.P., Feng H.M., Walker D.H. (2002). Current status of immune mechanism of killing of intracellular microorganisms. FEMS Microbiol. Lett..

[79] Yung S.C., Murphy P.M. (2012). Antimicrobial chemokines. Front. Immunol..

[80] Gomes R.N., Teixeira-Cunha M.G., Figueiredo R.T., Almeida P.E., Alves S.C., Bozza P.T., Bozza F.A., Bozza M.T., Zimmerman G.A., Castro-Faria-Neto H.C. (2013). Bacterial clearance in septic mice is modulated by MCP-1/CCL2 and nitric oxide. Shock.

[81] Depaolo R.W., Lathan R., Rollins B.J., Karpus W.J. (2005). The chemokine CCL2 is required for control of murine gastric *Salmonella enterica* infection. Infect. Immun..

[82] Collin M., Linge H.M., Bjartell A., Giwercman A., Malm J., Egesten A. (2008). Constitutive expression of the antibacterial CXC chemokine GCP-2/CXCL6 by epithelial cells of male reproductive tract. J. Reprod. Immunol..

[83] Valdivia-Silva J., Medina-Tayamo J., Gracia-Zepeda E.A. (2015). Chemokine-derived peptides: Novel antimicrobial and antineoplastic agents. Int. J. Mol. Sci..

[84] Söbirk S.K., Mörgelin M., Egesten A., Bates P., Shannon O., Collin M. (2013). Human chemokines as antimicrobial peptides with direct parasiticidal effect on *Leishmania mexicana* in vitro. PLoS ONE.

[85] Ioannidis L.J., Nie C.Q., Hansen D.S. (2014). The role of chemokines in severe malaria: More than meets the eye. Parasitology.

[86] Domingo-Gonzalez R., Prince O., Cooper A., Khader S.A. (2016). Cytokines and Chemokines in *Mycobacterium tuberculosis* Infection. Microbiol. Spectr..

[87] Zhou R.P., Wu X.S., Xie Y.Y., Dai B.B., Hu W., Ge J.F., Chen F.H. (2016). Functions of interleukin-34 and its emerging association with rheumatoid arthritis. Immunology.

[88] Truong A.D., Hong Y., Lee J., Lee K., Kil D.Y., Lillehoj H.S., Hong Y.H. (2018). Interleukin-34 Regulates Th1 and Th17 Cytokine Production by Activating Multiple Signaling Pathways through CSF-1R in Chicken Cell Lines. Int. J. Mol. Sci..

[89] Lin X., Luo H., Yan X., Song Z., Gao X., Xia Y., Zhang L., Yin Y., Cao J. (2018). Interleukin-34 ameliorates survival and bacterial clearance in polymicrobial sepsis. Crit. Care Med..

[90] Mikalsen A.B., Torgersen J., Aleström P., Hellemann A.L., Koppang E.O., Rimstad E. (2004). Protection of atlantic salmon Salmo salar against infectious pancreatic necrosis after DNA vaccination. Dis. Aquat. Organ..

[91] Kanellos T., Sylvester I.D., D’Mello F., Howard C.R., Mackie A., Dixon P.F., Chang K.C., Ramstad A., Midtlyng P.J., Russel P.H. (2006). DNA Vaccination Can Protect *Cyprinus Carpio* Against Spring Viraemia of Carp Virus. Vaccine.

[92] Caipang C.M., Takano T., Hirono I., Aoki T. (2006). Genetic Vaccines Protect Red Seabream, *Pagrus Major*, Upon Challenge With Red Seabream Iridovirus (RSIV). Fish Shellfish Immunol..

[93] Pasnik D.J., Smith S.A. (2006). Immune and histopathologic responses of DNA-vaccinated hybrid striped bass *Morone Saxatilis X M. Chrysops* after acute *Mycobacterium marinum* infection. Dis. Aquat. Organ..

[94] Chen J., Tan W., Wang W., Hou S., Chen G., Xia L., Lu Y. (2019). Identification of common antigens of three pathogenic Nocardia species and development of DNA vaccine against fish nocardiosis. Fish Shellfish Immunol..

[95] Munang’andu H.M., Evensen Ø. (2019). Correlates of protective immunity of fish vaccines. Fish Shellfish Immunol..

[96] Cai S.H., Huang Y.C., Lu Y.S., Wu Z.H., Wang B., Tang J.F., Jian J.C. (2013). Expression and immunogenicity analysis of accessory colonization factor A from *Vibrio alginolyticus* strain HY9901. Fish Shellfish Immunol..

[97] Xing J., Xu H., Wang Y., Tang X., Sheng X., Zhan W. (2017). Protective efficacy of six immunogenic recombinant proteins of *Vibrio anguillarum* and evaluation them as vaccine candidate for flounder (*Paralichthys olivaceus*). Microb. Pathog..

[98] Xing J., Xu H., Wang Y., Tang X., Sheng X., Zhan W. (2017). Identification of immunogenic proteins and evaluation of four recombinant proteins as potential vaccine antigens from *Vibrio anguillarum* in flounder (*Paralichthys olivaceus*). Vaccine.

