# Supplementary material for: Interleukin 34 Serves as a Novel Molecular Adjuvant against Nocardia Seriolae Infection in Largemouth Bass (Micropterus Salmoides)
Source: Vaccines (Basel). 2020 Mar 28;8(2):151. doi: 10.3390/vaccines8020151 (PMC7349345; doi:10.3390/vaccines8020151)
Supplement: Supplementary file 1 [file vaccines-08-00151-s001.pdf]

vaccines

MDPI

Supplementary Materials

Interleukin 34 serves as a novel molecular adjuvant against *Nocardia seriolae* infection in largemouth bass (*Micropterus salmoides*)

Huy Hoa Hoang, Pei-Chi Wang, Shih-Chu Chen

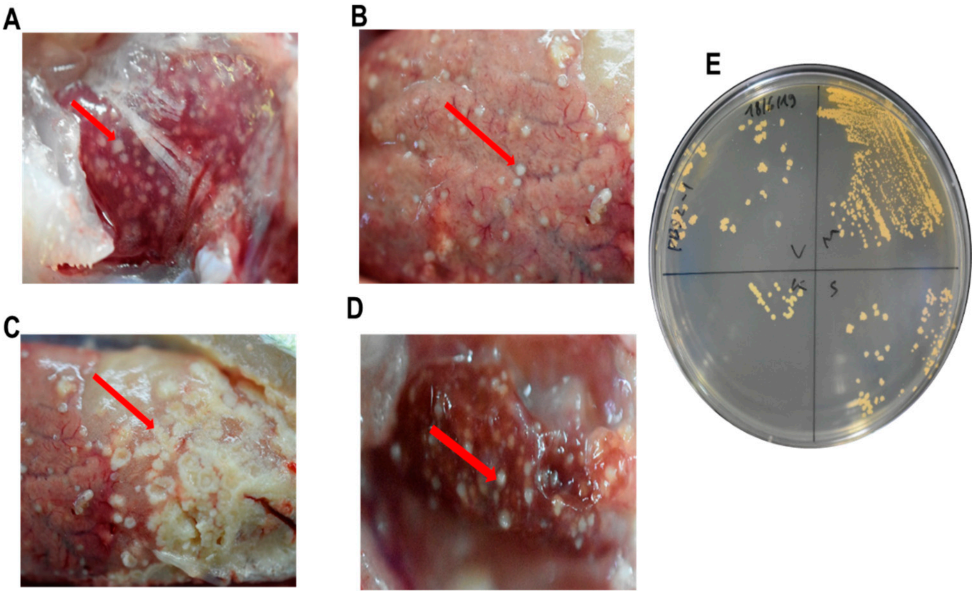

Figure S1. Multiple white nodules found in different organs of dead fish and re-isolation of *N. seriolae*. **(A)** Head kidney; **(B)** Liver; **(C)** Mesentery; **(D)** Spleen; **(F)** Pure colonies of *N. seriolae* growth in BHI-agar. Red arrow indicated the white nodules

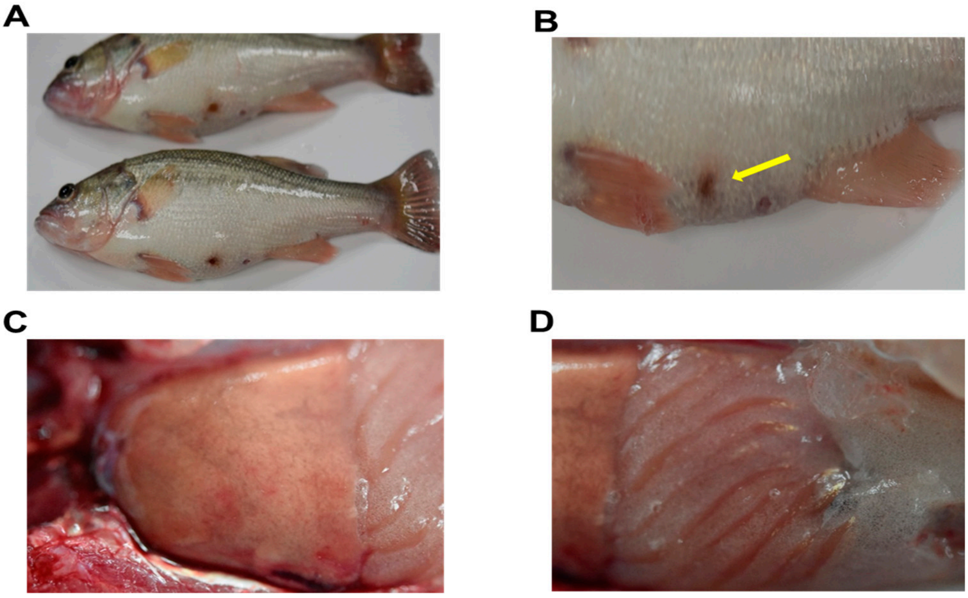

Figure S2. Pathological signs of survival fish in pcIL-34 vaccinated group. **(A)** Side view of fish; **(B)** No ulcer found in ventral side of fish – injection site (yellow arrows); **(C)** Liver; **(D)** Mesentery.

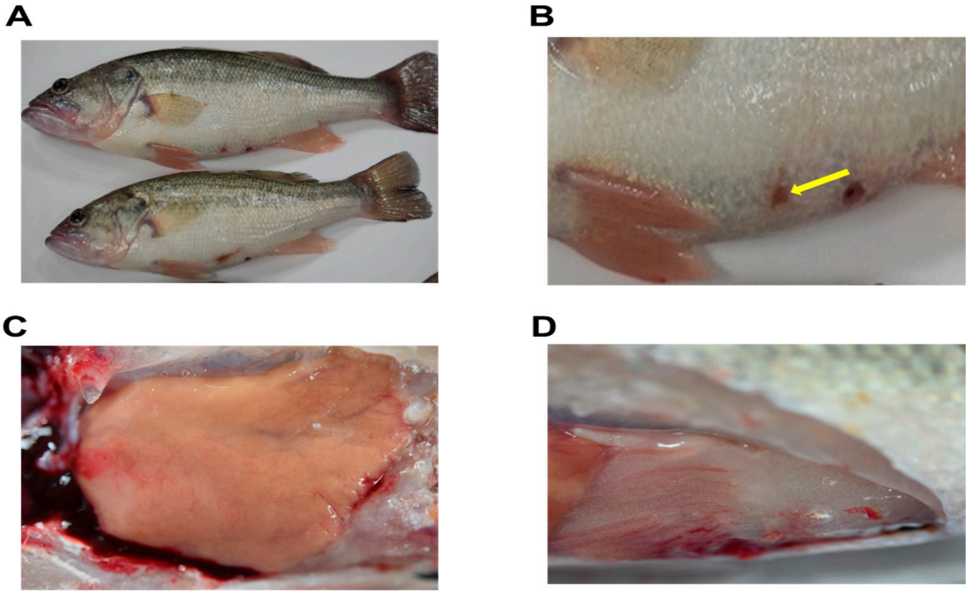

Figure S3. Pathological signs of survival fish in pcHrp1 vaccinated group. **(A)** Side view of fish; **(B)** No ulcer found in ventral side of fish – injection site (yellow arrows); **(C)** Liver; **(D)** Mesentery.

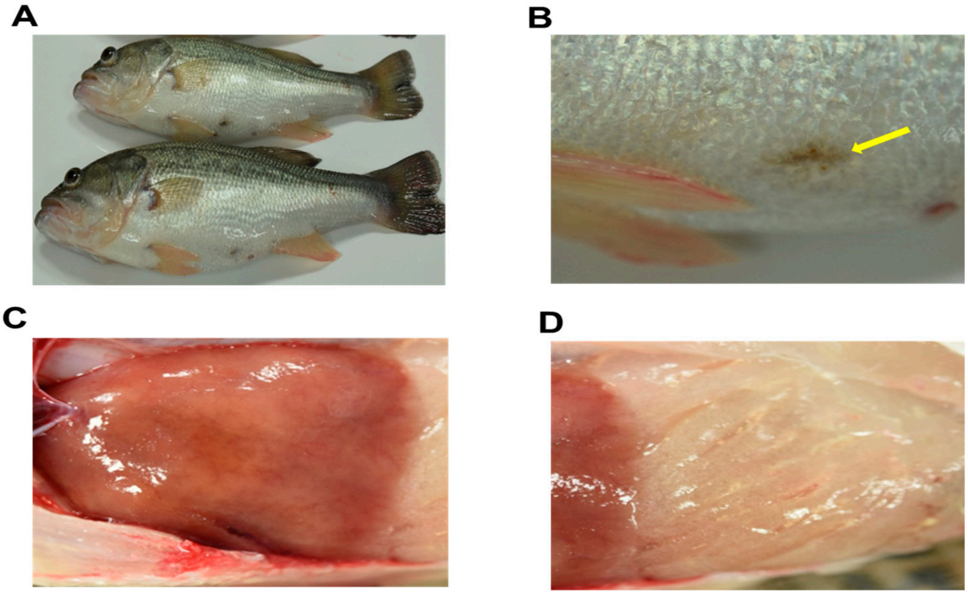

Figure S4. Pathological signs of survival fish in pcHrp1 + pcIL-34 vaccinated group. **(A)** Side view of fish; **(B)** No ulcer found in ventral side of fish – injection site (yellow arrows); **(C)** Liver; **(D)** Mesentery.

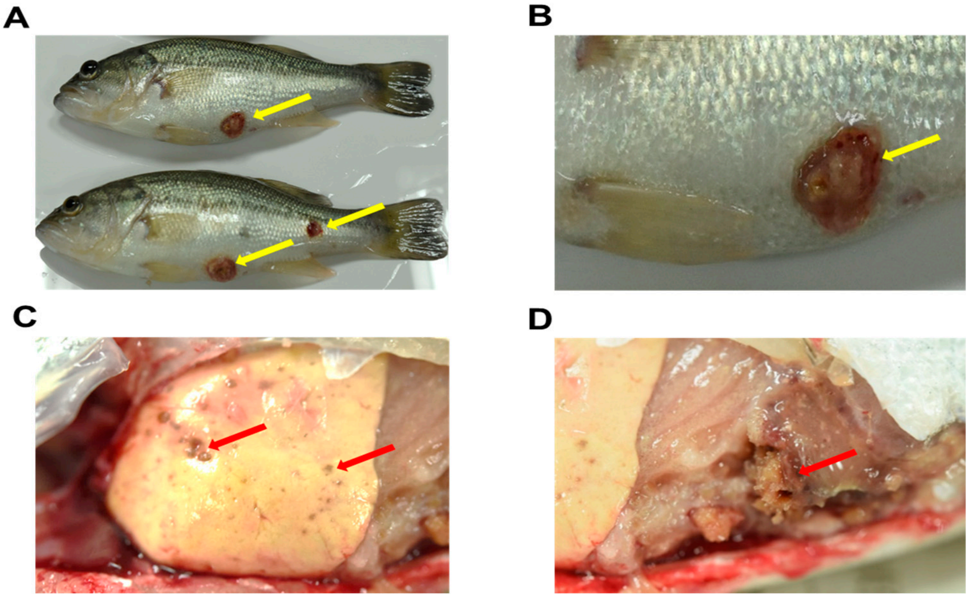

Figure S5. Pathological signs of survival fish in PBS injected group. **(A)** Side view of fish with ulcer at injection site (yellow arrows); **(B)** Ulcer found in ventral side of fish – injection site (yellow arrows); **(C)** Nodules found in liver (red arrow) ; **(D)** Nodule found in mesentery (red arrow).

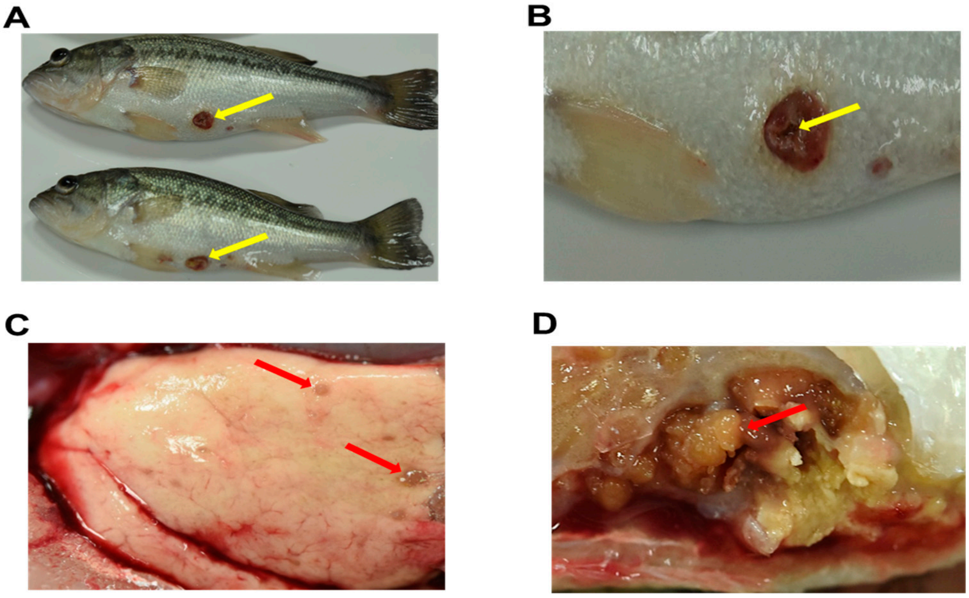

Figure S6. Pathological signs of survival fish in pcDNA3.1 injected group. **(A)** Side view of fish with ulcer at injection site (yellow arrows); **(B)** Ulcer found in ventral side of fish – injection site (yellow arrows); **(C)** Nodules found in liver (red arrow) ; **(D)** Nodule found in mesentery (red arrow).

© 2020 by the authors. Licensee MDPI, Basel, Switzerland. This article is an open access article distributed under the terms and conditions of the Creative Commons Attribution (CC BY) license (http://creativecommons.org/licenses/by/4.0/).
